# Domain acquisition by class I aminoacyl-tRNA synthetase urzymes coordinated the catalytic functions of HVGH and KMSKS motifs

**DOI:** 10.1093/nar/gkad590

**Published:** 2023-07-20

**Authors:** Guo Qing Tang, Jessica J H Elder, Jordan Douglas, Charles W Carter

**Affiliations:** Department of Biochemistry and Biophysics, University of North Carolina, Chapel Hill, NC 27599-7260, USA; Department of Biochemistry and Biophysics, University of North Carolina, Chapel Hill, NC 27599-7260, USA; Department of Biochemistry and Biophysics, University of North Carolina, Chapel Hill, NC 27599-7260, USA; Department of Physics, The University of Auckland, New Zealand; Department of Biochemistry and Biophysics, University of North Carolina, Chapel Hill, NC 27599-7260, USA

## Abstract

Leucyl-tRNA synthetase (LeuRS) is a Class I aminoacyl-tRNA synthetase (aaRS) that synthesizes leucyl-tRNA^leu^ for codon-directed protein synthesis. Two signature sequences, HxGH and KMSKS help stabilize transition-states for amino acid activation and tRNA aminoacylation by all Class I aaRS. Separate alanine mutants of each signature, together with the double mutant, behave in opposite ways in *Pyrococcus horikoshii* LeuRS and the 129-residue urzyme ancestral model generated from it (LeuAC). Free energy coupling terms, Δ(Δ*G*^‡^), for both reactions are large and favourable for LeuRS, but unfavourable for LeuAC. Single turnover assays with ^32^Pα-ATP show correspondingly different internal products. These results implicate domain motion in catalysis by full-length LeuRS. The distributed thermodynamic cycle of mutational changes authenticates LeuAC urzyme catalysis far more convincingly than do single point mutations. Most importantly, the evolutionary gain of function induced by acquiring the anticodon-binding (ABD) and multiple insertion modules in the catalytic domain appears to be to coordinate the catalytic function of the HxGH and KMSKS signature sequences. The implication that backbone elements of secondary structures achieve a major portion of the overall transition-state stabilization by LeuAC is also consistent with coevolution of the genetic code and metabolic pathways necessary to produce histidine and lysine sidechains.

## Graphical Abstract

**Graphical Abstract:**
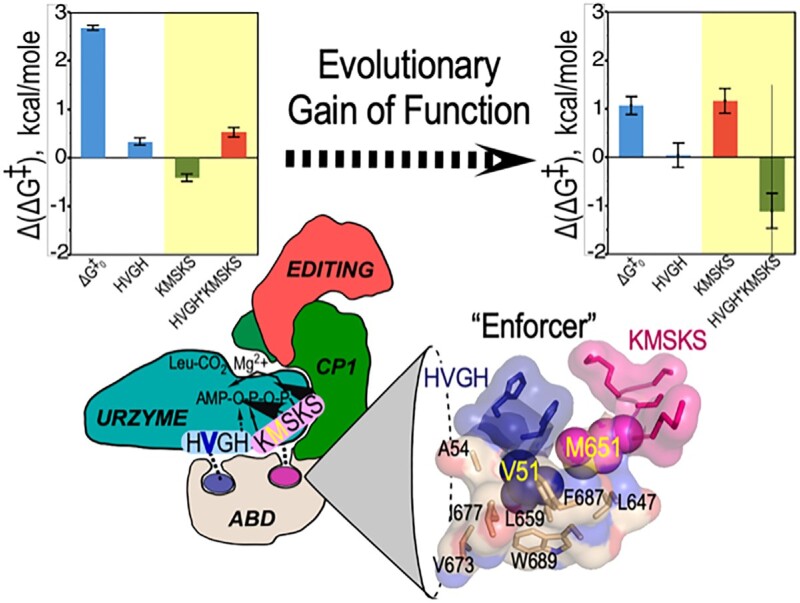
Thermodynamic cycle analysis of the free energy contributions of the HxGH and KMSKS catalytic signatures in Leucyl-tRNA synthetase and its 129-residue urzyme identify dramatic changes in the relative contributions of the KMSKS sequence and the HVGH*KMSKS two-way interaction. Structural analysis shows that a conserved network of non-polar packing in the anticodon binding domain functions as an “Enforcer” to impose coupled behavior in the full-length enzyme.

## INTRODUCTION

**Figure 1.**
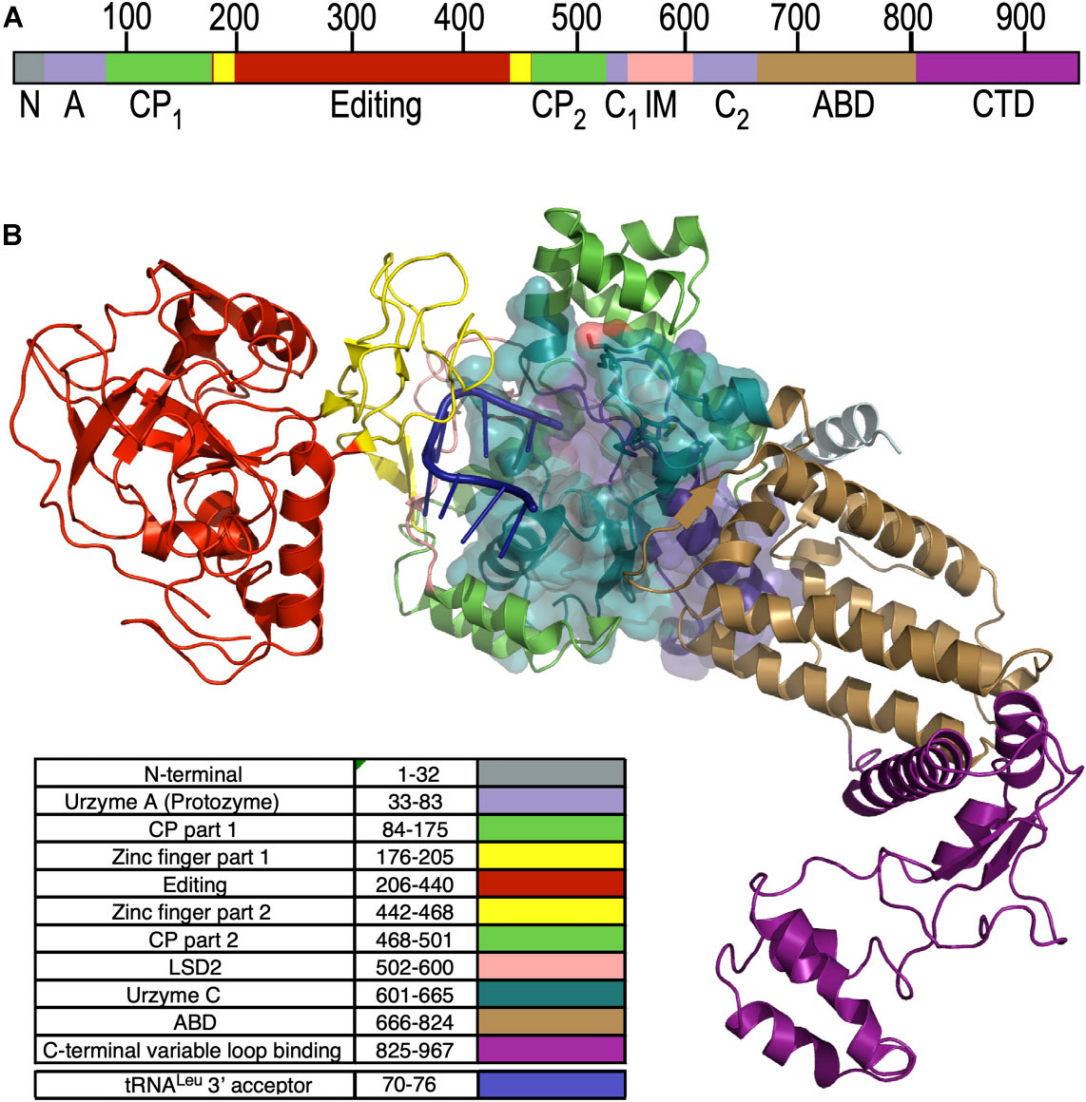
Relation of LeuAC urzyme to full-length *P. horikoshii* LeuRS. Colors are consistent between A and B. (**A**) Linear schematic of LeuRS domains. N,N-terminal helical domain; A, Protozyme (ATP-binding site); CP, connecting peptide, a nested insertion containing the editing domain; Editing, Editing domain; C_1_, second fragment of the urzyme (amino acid and tRNA binding). LSD_2_, Leucine specific domain 2 (11), an insertion module before second crossover; C_2_, C-terminal fragment of the urzyme (pyrophosphate binding); ABD, anticodon-binding domain; CTD, C-terminal domain that binds to the tRNA^Leu^ variable loop. (**B**) Three-dimensional cartoon based on PDB ID 1WC2 showing the LeuAC urzyme (surface embedded into the full-length enzyme and the arrangement of modules acquired during the specialization of LeuRS from other Class I aaRS. A76 of the tRNA^Leu^ acceptor stem inserts into the active sites of both LeuRS and LeuAC, bisecting the protozyme (blue) and urzyme part C (C_2_), the second half of the urzyme. Insertions into a loop connecting the protozyme and C2 are all nested, one within the next, forming the complete CP. In contrast, C-terminal additions are serial.

Class I aminoacyl-tRNA synthetases (aaRS) are one of two enzyme superfamilies that, together with their cognate tRNAs, translate the genetic code (1). They perform that function by activating cognate amino acids at the expense of the two high-energy bonds in ATP and transferring the activated aminoacyl group from the 5′ position of AMP to the tRNA 3′-terminal ribose. Eleven contemporary Class I aaRS families are responsible for the hydrophobic amino acids (isoleucine, valine, leucine, methionine), together with the larger homologs of the acidic (glutamate), amide (glutamine), and aromatic (tryptophan, tyrosine) amino acids, as well as arginine and cysteine. Some archaea and bacteria also possess a Class I lysyl-tRNA synthetase (2), which in most organisms is a Class II enzyme. All Class I aaRS share the same three domains (Figure 1): a Rossmann dinucleotide-binding catalytic domain, variable-length insertions between the two crossover connections of the Rossmann fold (CP) (3,4) including an embedded editing domain in larger Class I enzymes, and an idiosyncratic anticodon-binding domain, ABD. The active site within the Rossmann fold is highly conserved across the superfamily (5). Moreover, that domain has been re-constructed without any of the other domains, and it retains all three functions—amino acid activation, retention of the activated amino acid, and transfer to cognate tRNA—necessary to implement a rudimentary form of coding. For these reasons, we have argued that such constructs, called ‘urzymes’(6), are excellent experimental models for studying the emergence and early evolutionary history of ancestral aaRS (7–10) and genetic coding generally.

Here, we utilize the leucyl-tRNA synthetase from *Pyrococcus horikoshii*, LeuRS (11) and its urzyme, LeuAC (12), to examine the evolutionary gain of function associated with acquiring the CP and ABD domains. We note that as *P. horikoshii* expresses the archaeal-like LeuRS, which is monomeric, LeuAC is also very likely monomeric. The location of its post-transfer editing domain within the extended connecting peptide (CP) insertion differs from that in eubacterial LeuRS (13). Construction of the LeuAC urzyme entailed removing the entire extended CP domain, which includes many acquired insertion modules (Douglas, J., Bouckaert, R., Carter, C.W.J. and Wills, P. (2023) Enzymic recognition of amino acids drove the evolution of primordial genetic codes. *Research Square* DOI 10.21203/rs.3.rs-2924681/v1) as well as the ABD and C-terminal domains (Figure 1). LeuAC is thus homologous in that respect to the corresponding urzyme excerpted from *Geobacillus stearothermophilus*, TrpAC (6,14). However, the mass deleted from the catalytic domain (377 amino acids) is ∼ five times more than were deleted (74 amino acids) from the TrpRS catalytic domain. That missing mass includes insertion modules that others have suggested were essential to aminoacylation (15). LeuAC required a modest number of sequence changes on the surface to stabilize the removal of the Connecting Peptide. Supplementary Figure S1 in the Supplementary Information for (12) summarizes these sequence changes.

Two consensus sequence motifs present in the Rossmann fold (HIGH and KMSKS) have been implicated multiple ways in catalysis of both amino acid activation and acyl-transfer to tRNA in many Class I aaRS. Their conservation across the superfamily has been an evolutionary curiosity, suggesting that they are central to catalysis. However, both histidine and lysine require complex biosynthetic pathways, and thus are unlikely to have been available for the earliest stages of codon-dependent translation (16–19).

The unexpectedly modest impact of mutating lysine residues to alanine in the LeuAC urzyme KMSKS signature (12) motivated us to examine the catalytic roles of both signatures in both LeuRS and LeuAC. An obvious way to do this is with a factorial design in which both histidines in the HxGH signature are also mutated to alanine separately and in combination. That combinatorial mutagenesis measures the energetic coupling between the two signatures. To our knowledge, and despite a landmark combinatorial mutagenesis of the KFGKT sequence in TyrRS (20–23), no one has examined the coordinated behavior of the two Class I catalytic signatures, even in a full-length Class I aaRS. Comparing that coupling in urzyme and full-length enzyme maps changes in the evolutionary time domain, hence measures the catalytic gain of function from integrating the domains deleted in the urzyme.

The comparison reinforces previous suggestions (24–27) that full-length Class I aaRS benefit from a significant intramolecular cooperativity that is absent from the corresponding urzymes. We identify side chain packing interactions between the hydrophobic side chains in each catalytic signature and a conserved cluster of nonpolar side chains in the anticodon-binding domain. Embedding the valine and methionine side chains of the LeuRS HVGH and KMSKS sequences into that cluster provides a structural rationale for their coordination during the catalytic cycle, consistent with their catalytic coupling in the full-length enzyme.

The anti-coupling between HVGH and KMSKS signatures in the urzyme has important implications for the emergence and early evolution of polypeptide catalysts. Surprisingly, the AVGA variant of the LeuAC urzyme benefits from both the wild type KMSKS signature and unfavorable coupling energy; it is thus the most active LeuAC variant and is actually more active at aminoacylation than the corresponding mutant of full-length LeuRS. Catalysis by aaRS urzymes does not require specific amino acid side chains that are highly conserved in the active sites of all Class I aaRS, thus significantly broadening the sequence space for primordial catalysts and creating substantive experimental support for Wong's proposal that the genetic code coevolved with the amino acid biosynthetic pathways (16,17).

Finally, the consistent response to combinatorial mutational changes evident from the thermodynamic cycle analysis, and provision of a structural rationale for differential intramolecular coupling in the full-length enzyme both also decisively confirm the authenticity of catalysis by aaRS urzymes.

## MATERIALS AND METHODS

### Expression and purification of LeuRS and LeuAC

The gene for *Pyrococcus horikoshii* (Ph) LeuRS was synthesized by Gene Universal and expressed from pET-11a in BL21-CodonPlus (DE3)-RIPL (Agilent). Cells were grown at 37°C and induced with 300 μM IPTG for 4 h then harvested and stored overnight at –20°C. The cell pellet was resuspended in 1× Ni-NTA buffer (20 mM Tris, pH 8.0, 300 mM NaCl, 10 mM imidazole, 5 mM β-ME) plus cOmplete protease inhibitor (Roche) and lysed by three 15k psi passes on an Avestin Emulsiflex. Cell debris was pelleted at 4°C 30 min 20k rpm. The soluble fraction was heated at 80°C for 30 min to denature native *Escherichia coli* proteins. The heated cell extract was then pelleted, and the soluble material was loaded on to an equilibrated Ni-NTA column. The column was washed with three volumes 1× Ni-NTA buffer, then protein was eluted in a stepwise fashion with imidazole concentrations of 40, 80, 100, 200, and 500 mM imidazole. The fractions containing the protein of interest were pooled and dialyzed overnight against 200 mM HEPES, pH 7.4, 450 mM NaCl, 100 mM KCl, 10 mM β-ME. The following day the dialyzed protein was concentrated, brought to 50% glycerol and stored at –20°C.

LeuAC was expressed as an MBP fusion from pMAL-c2x in BL21Star(DE3) (Invitrogen). Cells were grown, induced, harvested, and lysed similarly to Ph LeuRS with the distinct difference of being resuspended in buffer (20 mM Tris, pH 7.4, 1 mM EDTA, 5 mM β-ME, 17.5% glycerol, 0.1% NP40, 33 mM (NH_4_)_2_SO_4_, 1.25% glycine, 300 mM guanidine hydrochloride) plus cOmplete protease inhibitor (Roche). LeuAC crude extract was then pelleted at 4°C 30 min 15k rpm to remove insoluble material. The extract was then diluted 1:4 with Optimal Buffer and loaded onto equilibrated Amylose FF resin (Cytiva). The resin was washed with five column volumes of buffer and the protein was eluted with 10 mM maltose in Optimal Buffer. Fractions containing protein were concentrated, brought to 50% glycerol and stored at –20°C. All protein concentrations were determined using the Pierce™ Detergent-Compatible Bradford Assay Kit (Thermo Scientific). Experimental assays were performed either with the intact MBP-LeuAC fusion protein or with samples cleaved by tobacco etch virus (TEV) protease, purified as described (28). Purity and cleavage efficiency was determined by running samples on PROTEAN® TGX (Bio-RAD) gels.

### Single turnover active-site titration assays

Active-site titration assays were performed as described (29,30). 3 μM of protein was added to 1x reaction mix (50 mM HEPES, pH 7.5, 10 mM MgCl_2_, 5 μM ATP, 50 mM amino acid, 1 mM DTT, inorganic pyrophosphatase, and α-labeled [^32^P] ATP) to start the reaction at 37°C for LeuAC and on ice, or slightly above 0°C for LeuRS. The α labeling position allowed us to follow time courses of ADP and AMP production, as well as for ATP consumption. Timepoints were quenched in 0.4 M sodium acetate 0.1% SDS and kept on ice until all points had been collected. Quenched samples were spotted on TLC plates, developed in 850 mM Tris, pH 8.0, dried and then exposed for varying amounts of time to a phosphor image screen and visualized with a Typhoon Scanner (Cytiva). ImageJ (31) was used to quantitate intensities of each nucleotide. The time-dependent of loss (ATP) or de novo appearance (ADP, AMP) of the three adenine nucleotide phosphates were fitted using the nonlinear regression module of JMP16PRO™ (SAS Institute, Cary NC) to equation (1) (29):


(1) \begin{eqnarray*} {\mathrm{Product}}\left( {\mathrm{calc}} \right) &=& {{\mathrm{A}}}^ {*} {\mathrm{exp}}\left( {{\mathrm{ - }}{{\mathrm{k}}}_{{\mathrm{chem}}}^{\mathrm{*}}{\mathrm{seconds}}} \right)\nonumber \\ && - \, {{\mathrm{k}}}_{{\mathrm{cat}}}^{\mathrm{*}}{\mathrm{seconds + C}}\end{eqnarray*}

where *k*_chem_ is the first-order rate constant, *k*_cat_ is the rate of turnover, *A* is the amplitude of the first-order process or the size of the ‘burst’, and *C* is an offset. The burst size is proportional to the number of active molecules responsible for producing a product in the first round of catalysis in the added catalyst. Eq. (1) was also used for convenience to fit the appearance of nucleotide products, AMP and ADP. In that case the roles of *A* and *C* are inverted, *C* being the burst size and *A* the offset and both rate constants have the opposite sign.

### Burst size estimation from fitted parameters in equation (1)

For [ATP] decay curves, the fitted *A* value estimates *n* directly as *n* = A*[ATP]/[Enzyme]. *C* gives *n* = (1 – *C*)*[ATP]/[enzyme]. For exponentially increasing concentrations of product, the situation reverses, and *n* = (1 – *A*) *[ATP]/[enzyme] and *n* = C*[ATP]/[enzyme]. The very small variance of multiple estimates and the internal self-consistency discussed in (RESULTS:Single turnover…) justify these approximations.

### tRNA^Leu^ aminoacylation assays

A plasmid encoding the *P. horikoshii* tRNA^Leu^ (TAG codon) was synthesized by Integrated DNA Technologies and used as template for PCR amplification of the tRNA and upstream T7 promoter and downstream Hepatitis Delta Virus (HDV) ribozyme. The PCR product was used directly as template for T7 transcription. Following a 4-hour transcription at 37°C the RNA was cycled five times (90°C for 1 min, 60°C for 2 min, 25°C for 2 min) to increase the cleavage by HDV. The tRNA was purified by urea PAGE and crush and soak extraction. The tRNA 2′–3′ cyclic phosphate was removed by treatment with T4 PNK (New England Biolabs) following the manufacturer's protocol. The tRNA was then phenol chloroform isoamyl alcohol extracted, filter concentrated, aliquoted, and stored at –20°C.

We determined the active fraction of tRNA^Leu^ by following extended acylation assays using full-length *P. horikoshii* LeuRS until they reached a plateau. That plateau value was 0.38, which we used to compute tRNA^Leu^ concentrations in assays with both LeuRS and LeuAC.

Aminoacylations were performed in 50 mM HEPES, pH 7.5, 10 mM MgCl_2_, 20 mM KCl, 5 mM DTT with indicated amounts of ATP and amino acids. Desired amounts of unlabeled tRNA—mixed with [α^32^P] A76-labeled tRNA for assays by LeuAC—were heated in 30 mM HEPES, pH 7.5, 30 mM KCl to 90°C for 2 min. The tRNA was then cooled linearly (drop 1°C/30 s) until it reached 80°C when MgCl_2_ was added to a final concentration of 10 mM. The tRNA continued to cool linearly until it reached 20°C.

### Data processing and statistical analysis

Phosphorimaging screens of TLC plates were densitometered using ImageJ (31). Data were transferred to JMP16PRO™ via Microsoft Excel (version 16.49), after intermediate calculations. We fitted active-site titration curves to Eq. (1) using the JMP16PRO™ nonlinear fitting module. *R*^2^ values were all >0.97 and most were >0.99.

Factorial design matrices in Supplementary Tables S1 and S2 were processed using the Fit model multiple regression analysis module of JMP16PRO™ Pro, using an appropriate form of equation (2) (32)


(2) \begin{eqnarray*}{{\mathrm{Y}}}_{{\mathrm{obs}}}{\mathrm{ = }}{{\mathrm{\beta }}}_{\mathrm{0}}{\mathrm{ + \Sigma }}{{\mathrm{\beta }}}_{\mathrm{i}}^{\mathrm{*}}{{\mathrm{P}}}_{\mathrm{i}}{\mathrm{ + \Sigma }}{{\mathrm{\beta }}}_{{\mathrm{ij}}}^{\mathrm{*}}{{\mathrm{P}}}_{\mathrm{i}}^{\mathrm{*}}{{\mathrm{P}}}_{\mathrm{j}}{\mathrm{ + \varepsilon }} \end{eqnarray*}

where *Y*_obs_ is a dependent variable, usually an experimental observation, *β*_0_ is a constant derived from the average value of *Y*_obs_, *β_i_* and *β_ij_* are coefficients to be fitted, *P_i,j_* are independent predictor variables from the design matrix, and ϵ is a residual to be minimized. All rates were converted to free energies of activation, Δ*G*^‡^ = –*RT*ln(*k*), before regression analysis because free energies are additive, whereas rates are multiplicative. For example, the activation free energy for the first-order decay rate in single-turnover experiments is Δ*G*^‡^*k*_chem_.

Multiple regression analyses of factorial designs exploit the replication inherent in the full collection of experiments to estimate experimental variances on the basis of *t*-test *P*-values, in contrast to the presenting error bars showing the variance of individual datapoints. Analyses reported here also entail triplet experimental replicates, which improve the associated analysis of variance.

## RESULTS

The experimental design matrix for this work is the 2^3^ factorial design that tests all possible combinations of HVGH → AVGA and KMSKS → AMSAS mutations in both full-length LeuRS and the LeuAC urzyme. The eight variants were constructed, expressed, and purified as described previously (12) and in MATERIALS AND METHODS. We assayed all variants for leucine activation by single turnover active-site titrations (Supplementary Table S1) and for aminoacylation of tRNA^Leu^ in triplicate (Supplementary Table S3). Active-site titration experiments provided suitable estimates of burst sizes for all variants of both LeuAC and LeuRS. However, first-order rate constants were too fast at room temperature to fit reliably for LeuRS variants. However, reactions on ice (at ∼0°C) were sufficiently slow to permit corresponding estimation of the mutational impact on leucine activation by LeuRS variants. The first section describes those results.

We then describe aminoacylation rates measured at saturating concentrations of leucine (12) and the highest achievable tRNA^Leu^ concentrations. The next section compares mutational impacts on leucine activation and tRNA^Leu^ aminoacylation by both LeuAC and LeuRS. A subsequent section places those results into the context of the evolutionary gain of function in evolving LeuRS from LeuAC. Finally, we provide a structural rationale for the enhanced energetic coupling between the two signatures in the fully-evolved enzyme.

### Single turnover experiments give consistent first-order rates for changes in ATP, ADP, and AMP for both LeuAC and LeuRS

Single turnover kinetic experiments, often called active-site titrations (AST) use substrate level amounts of enzyme. They measure the size of the burst corresponding to the first round of catalysis and are therefore the method of choice to estimate fractional activities of enzyme variants (29,33) and normalizing enzyme concentrations when computing *k*_cat_ values.

**Figure 2.**
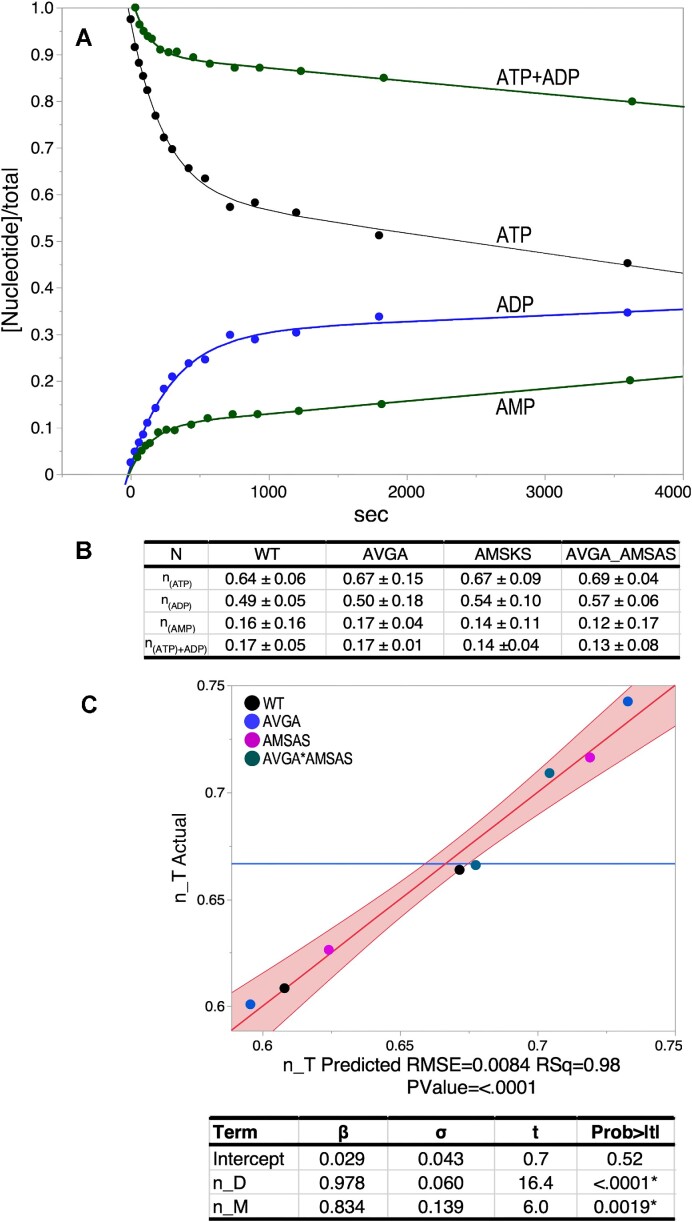
N-value determination for the four LeuAC variants. (**A**) Single turnover time courses for the three adenine nucleotides. The green ATP + ADP curve represents the ATP consumption after accounting for ADP production and is almost a reflection of the (green) curve for AMP production. Thus, it likely represents *in situ* formation of leu-5′AMP. (**B**) Averaged n-values determined from the two physical constants, A (the amplitude of the burst) and C (the offset), with their standard deviations obtained for each variant by fitting to Eq. (1) as described in Materials and Methods. (**C**) Multivariate regression analysis of *n*-values averaged in **B** confirm that n-values for the appearance of ADP and AMP account almost exactly for ATP consumption. Solving the simultaneous equations [*n*_*T_i_* = Σ_*i*,variants_ (β_D_(*C* + *n*_D) + β_M_(*n*_M) + ϵ)] by least squares using JMP16PRO gave the table of values underneath the graph. Here, and elsewhere, the true value of the regression line has a 95% chance of falling within the red boundaries.

AST assays performed here to estimate the active fractions in all variants provide supplemental evidence that mutational impacts are comparable in both leucine activation and aminoacylation by LeuAC. We uncovered an unexpected phenomenon when we followed the time courses for all three adenine nucleotides in AST assays of aaRS urzymes (Figure 2). ATP consumption is accompanied by ADP production (12), in addition to the expected products, aminoacyl-5′AMP and AMP, which is produced by product release and hydrolysis. Prior to analyzing kinetic data for the thermodynamic cycles presented here, we studied these curves in detail to assure a consistent interpretation.

Quantitation of the LeuAC active-site titration curves for the three adenine nucleotides (Figure 2A) confirms first that they are additive; *n*-values (Figure 2B) from ATP consumption are quantitative sums of those for ADP and AMP produced (Figure 2C). LeuAC variant *n*-values from fitting *C* and *A* in Eq. (1) agree with a fractional error of ∼0.09. ATP consumption <*n*_T> values range from 0.64 to 0.69 for the different combinatorial variants. <*n*_D > values (ADP) range from 0.49 to 0.57. <*n*_M> values (AMP) range from 0.13 to 0.17. In each case, *n*_T = *n*_D + *n*_M within 2% (i.e. intercepts are ∼0; slopes are both ∼1). These data furnish a self-consistent picture of multiple catalyzed reactions occurring in the first round of catalysis (Supplementary Table S2).

LeuAC variant-specific column vectors in Figure 2B are quite strictly parallel; all six correlation coefficients have *R*^2^ values > 0.99. Thus, although combinatorial mutagenesis changes the active fractions, it has no effect on the relative magnitudes of the four different n-values. Strict linear dependence means that, for the purpose of normalizing the active fractions of different variants in analyzing aminoacylation rates, any of the n-values would be equally suitable. For the present purpose, we chose to normalize acylation rates using the *n*_T values, to estimate the minimum rate enhancements of acylation by full length LeuRS, relative to LeuAC.

The preceding analysis provides additional circumstantial evidence supporting our earlier proposal that ADP production arises from successive phosphorylations of bound leucyl-5′AMP. That proposal was originally based on two observations (12). (i) AMP production exhibits a first order rate early in the titration, followed by a sharp increase in the steady state rate (see Supplementary Figure S2(c) in (12). (ii) The 3D structure of the LeuAC active-site configuration provides alternative ATP-binding sites that place the γ-phosphate group into suitable positions to phosphorylate the α-phosphate of the bound adenylate first to ADP and then to ATP (see Figure 9 of (12)). If those two phosphorylation reactions regenerated ATP, it would promote bound Leu-5′AMP to a transition-state-like configuration. That would be consistent with the relatively independent burst sizes and relative proportions of ADP and AMP.

### Combinatorial mutants of HIGH and KMSKS catalytic signatures in LeuRS reduce its catalytic proficiency for tRNA^Leu^ acylation to that of LeuAC and its mutational variants

**Figure 3.**
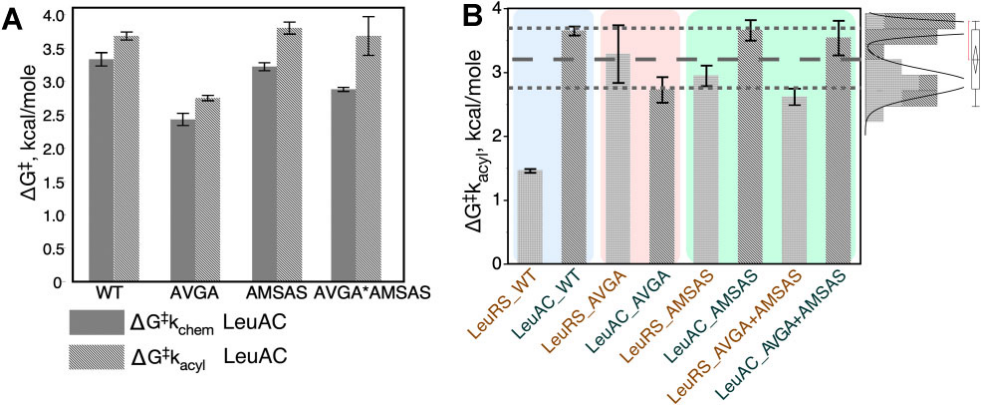
Individual transition-state stabilization free energies, in kcal/mole, for each LeuRS variant identified along the x axis. Error bars are two-way 95% confidence levels from triplicate determinations. (**A**) Transition stabilization free energies, Δ*G*^‡^*k*_chem_ and Δ*G*^‡^*k*_acyl_ represent relative rate accelerations. All LeuAC variants show comparable changes in amino acid activation and tRNA^Leu^ aminoacylation. TS stablizations are greater for activation than for acylation, as expected from differences in the corresponding uncatalyzed rates (59). (**B**) LeuRS variants are generally stronger catalysts than LeuAC variants, with the largest gap being between variants with wild type sequences (blue panel). AVGA variants have inverted rates, (rose panel) because the AVGA LeuAC variant has greater activity than the corresponding full-length LeuRS variant. Finally, AMSAS and double mutant variants have reduced activity in LeuAC and increased activity in LeuRS, relative to the corresponding AVGA variants (green panel). Distributions in the upper right show all 21 measurements for variants excepting WT LeuRS. Remarkably, the AVGA LeuAC mutant falls within the group of full-length variants that are more active than the other three LeuAC variants. Grey dashed lines identify a significant subset who's mean Δ*G*^‡^*k*_acyl_, ∼3.2 kcal/mol, is significantly greater than that of WT LeuRS. Mutational variants of LeuRS, excepting AVGA, are nonetheless also better catalysts than those of corresponding LeuAC. This significant variation is difficult to interpret without thermodynamic cycle analysis.

Supplementary Table S4 summarizes the experimental data for aminoacylation of tRNA^Leu^. A histogram of transition-state stabilization free energies for tRNA^Leu^ aminoacylation, Δ*G*^‡^*k*_acyl_, for all eight variants (Figure 3) reveals that the two catalytic signatures make profound contributions to the catalytic proficiency of the full-length enzyme. The distributions in the upper right of Figure 3B illustrate that Δ*G*^‡^ measurements for all LeuRS mutational variants have values comparable to those of the wild type Urzyme LeuAC and its corresponding mutations. Rather than forming a separate distribution in the neighborhood of the WT LeuRS, as observed for LeuAC variants, any mutation compromises all LeuRS variants to levels of the LeuAC urzyme and its combinatorial mutants. Active-site mutations therefore have severe effects on the full-length enzyme, but substantially smaller effects on the urzyme. Thus, neither wild-type urzyme active site nor any full-length LeuRS variant can achieve a comparable rate enhancement chemistry to that of the full-length enzyme.

AST assays (Figure 2) furnish values of the internal first-order rate constant for first-round catalysis of leucine activation. Using these values, we can compare the impacts of mutation on the activation reaction. Figure 3A compares transition-state stabilization free energies of the four LeuAC variants in the two successive steps of tRNA^Leu^ biosynthesis. Qualitatively, the main difference between them is that all variants have lower Δ*G*^‡^ for activation than for aminoacylation. The AVGA mutant is markedly faster than any of the others, and the AMSAS variant is slowest by a small margin over the double mutant. These differences are easier to appreciate when transformed by estimating parameters for the corresponding thermodynamic cycle [Eq. (2)].

### Thermodynamic cycle analysis emphasizes the gain of function resulting from ABD and CP domain acquisition

The factorial design matrices (Supplementary Tables S1, S2) enable us to attribute a magnitude and statistical significance of free energy contributions to transition-state stabilization for intrinsic effects of each signature sequence and the synergy between them. For this purpose we use a construct known as a thermodynamic cycle, advocated by Jencks (34) and popularized by Horovitz and Fersht (35). As originally formulated, construction of a thermodynamic cycle entails computing differences observed in activation free energies, Δ(Δ*G*^‡^), at each corner of the implicit polygon of the factorial design. For a double mutant cycle, that involves a square with WT enzyme, the two single mutants, and the double mutant at the corners of a square (see Supplementary §II and Supplementary Figure S1).

We showed (26) that in practice it is more convenient to use multiple regression methods to estimate the coefficients in Eq. (2). When values of the experimental error, ϵ, are small and the regression model explains a high (≥0.9) portion of the variation in experimental data points, a thermodynamic cycle is equivalent to changing the coordinate system of the experimental free energies in Figure 3. Supplementary §II examines this equivalence in more detail, and Supplementary §III outlines how regression coefficients for main effects and lower-order interactions depend on where along the highest-order interaction they are evaluated.

**Figure 4.**
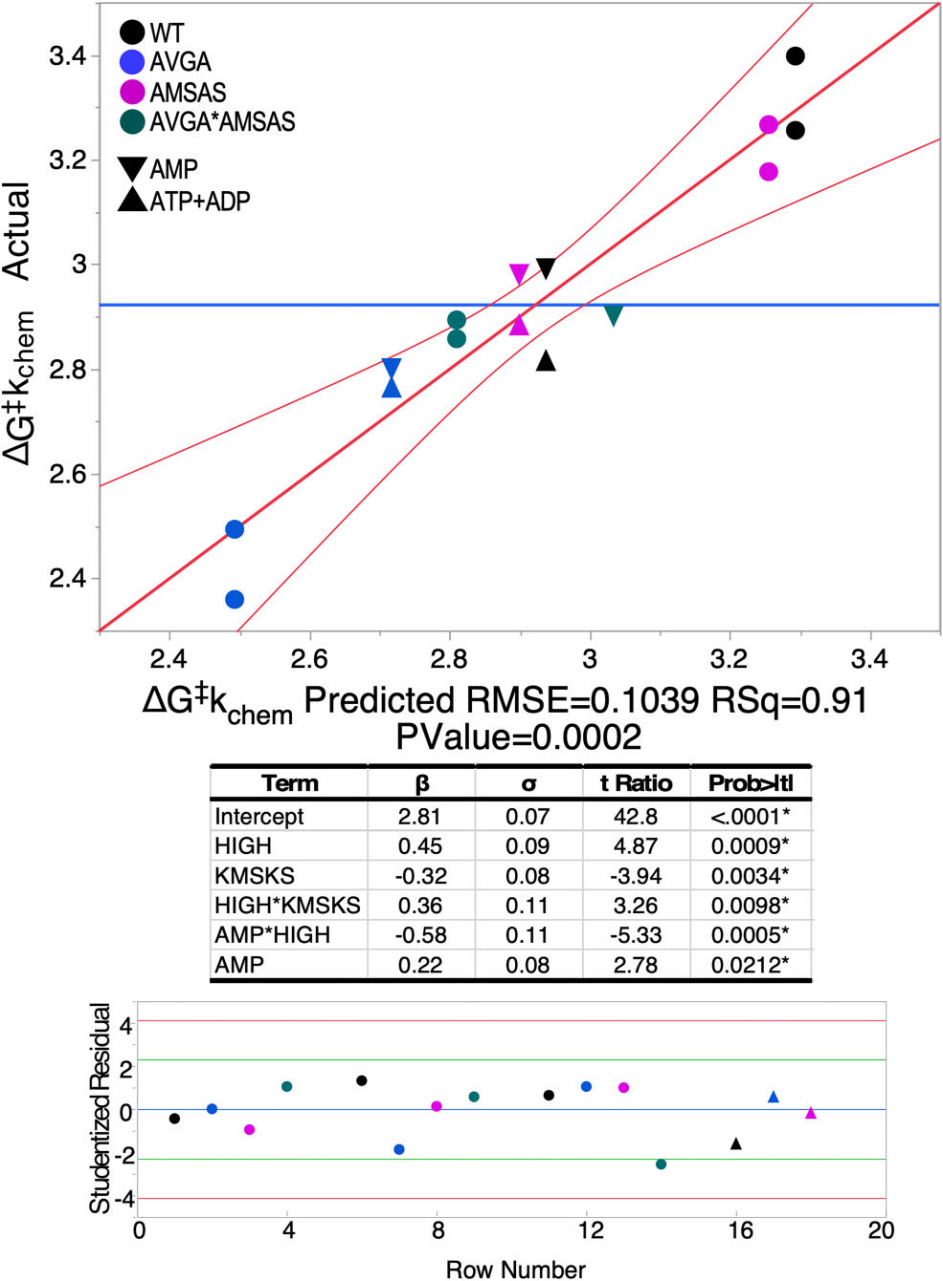
Multiple regression model for the activation free energies, Δ*G*^‡^*k*_chem_ for the internal first-order rate constant, *k*_chem_, for all LeuAC variants for time courses in Figure 1A. Circles represent ATP consumption and ADP production; triangles represent the two alternative measurements for AMP production. β-coefficients are in kcal/mole. The coloring scheme for variants is given in the upper left-hand corner. Studentized residuals (ϵ_St_, bottom) are all –4.0< ϵ_St_< 4.0 confirming the absence of outliers.

The regression model for the thermodynamic cycle for Δ*G*^‡^_kchem_ from first-order rates, for all three nucleotides either consumed or produced in amino acid activation (Figure 4) reveals several notable details. First, the distribution of Studentized residuals associated with the contributing data points (the prediction error, ϵ, divided by its adjusted standard error) lies between –4 ≤ 4, so there are no outliers. Second, there are five significant predictors listed in the table. Their Student t-test probabilities are all ≤0.02, hence are statistically significant. Third, because neither ATP nor ADP are significant predictors of the first-order rate, there is no significant difference between rates for ATP consumption and ADP formation for the four variants. AMP production, on the other hand, is significantly different, and the AMP*HVGH β-coefficient means that it depends on whether the HxGH sequence is native or mutant. We return to this point after considering mutational effects on tRNA^Leu^ acylation.

**Figure 5.**
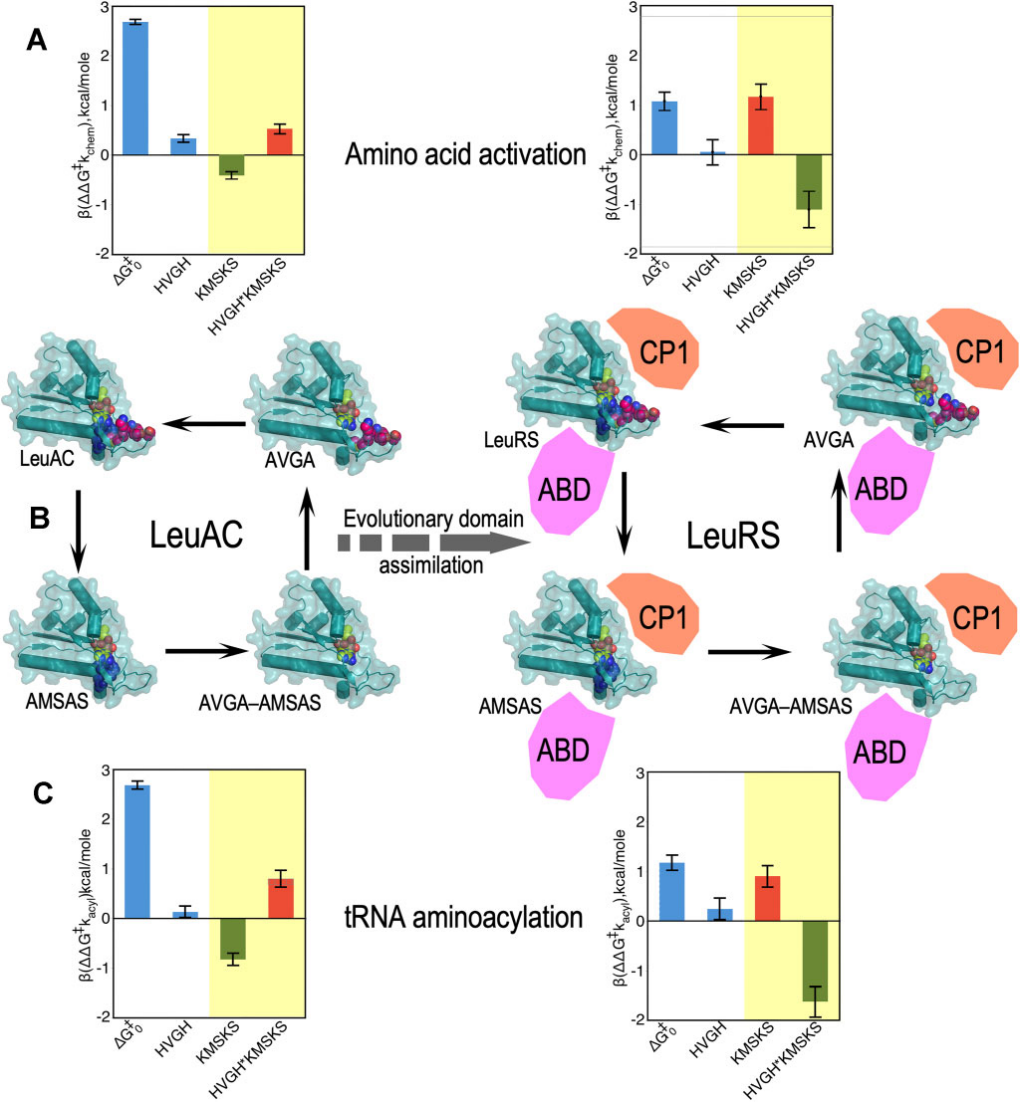
Thermodynamic cycles for LeuAC (left) and LeuRS showing amino acid activation (top) and tRNA aminoacylation (bottom). Data in Figure 3B and Supplementary Table S3 were transformed via Eqn. (2) into multiple regression coefficients, (*β_i_, β*_*ij*_), to determine successive changes in activation free energy, (Δ*G*^‡^*k*_chem_, (**A**) Δ*G*^‡^*k*_acyl_, **C**), for successive mutations shown by single-headed arrows around the square in (**B**) Error bars are derived from the estimated standard deviation of the (*β_i_, β*_*ij*_) values along the x-axes in A and C. Intrinsic effects of LeuAC and LeuRS catalysts and their HVGH signatures are similar in sign and relative magnitude (white panels). KMSKS and two-way HVGH*KMSKS Δ(Δ*G*^‡^) values however behave radically differently with and without the anticodon-binding (ABD) and CP domains (yellow panels). The two-way synergy between the two signatures (HVGH*KMSKS) changes from anti-cooperative and unfavourable in LeuAC (red bar) to strongly cooperative and favourable (green bar) in LeuRS, increasing transition-state stabilization for activation by ∼2 kcal/mol and aminoacylation of tRNA^Leu^ by ∼2.6 kcal/mol. Cartoons suggest the acquisition of CP and ABD domains in full-length LeuRS. The thick dashed arrow represents our interpretation that the domain acquisition is responsible for in the different histograms from LeuAC to LeuRS, hence representing evolutionary time. Note that LeuRS k_chem_ values were measured on ice and underestimate the values at 37°C.

First-order rate constants from AST, *k*_chem_, are independent of enzyme concentrations, and so do not require normalization for active fraction before converting to free energies for multiple regression analysis to construct thermodynamic cycles. Figure 4 highlights the statistical quality of the regression model (Figure 5 omits the corresponding models). Figure 5 displays histograms of the β-coefficients for the acylation reaction in kcal/mol, with error bars denoting the 95% confidence levels. Arrows in Figure 5B form cycles showing that the net free energy change of the successive steps total zero. The difference between left and right vertical arrows must equal that between top and bottom arrows, and is equal to the free energy, Δ(Δ*G*^‡^) for the HVGH*KMSKS interaction.

The most striking feature in Figure 4 is that samples at the extremes (the black dots = WT; the blue dots = AVGA) are opposite of what might be expected. The AVGA mutant is, by ∼ 1 kcal/mol, the most active catalyst in leucine activation and the WT variant is least active. Moreover, although the sign of the β_KMSKS_ coefficient is negative, signifying that the wild-type lysine residues favor catalysis, the synergy between it and the HIGH sequence, β_HVGH_*_KMSKS_, is positive. Thus, when it comes to stabilizing the transition state for the internally catalyzed reaction, the two signatures actually work against one another. That, and the positive β_HIGH_ value, account for the superiority of the AVGA variant, which is ∼5 times more active that wild-type LeuAC.

Figure 5 compares thermodynamic cycles (Figure 5B) for leucine activation (Figure 5A) and tRNA^Leu^ aminoacylation (Figure 5C) by LeuAC and LeuRS. Δ*G*^‡^_0_ is the mean activation free energy for all four variants of the respective catalysts; the remaining bars represent the additional transition-state stabilization (β < 0) or destabilization (β > 0) attributable to wild type HVGH, KMSKS, sequences and their two-way interaction or synergy. β coefficients for the main effects of the two mutations and their interaction tell us much about how the two catalytic signatures impact LeuAC catalysis. Wild-type residues in each location have opposite effects. Wild-type lysine residues in the KMSKS signature enhance catalysis modestly (ΔG‡KMSKS = –0.32 kcal/mole) compared to alanine mutants. Wild-type histidine residues in the HVGH signature actually reduce catalysis by a comparable amount (positive ΔG‡HVGH = 0.45 kcal/mol). The interaction term is also positive (ΔG‡HVGH*KMSKS = 0.36 kcal/mol), so the wild-type lysine residues in the KMSKS signature intensify the negative impact of the native HVGH sequence, and seemingly paradoxically, the presence of wild type KMSKS intensifies catalysis by mutant AVGA sequence.Combinatorial mutagenesis has comparable impacts on leucine activation and tRNA^Leu^ aminoacylation by LeuAC.

The KMSKS and HVGH*KMSKS effects behave very differently in full-length LeuRS. The inversion of these effects from left to right represents catalytic consequences of adding the anticodon binding domain and CP (as well as CP2) to the LeuAC urzyme. That consequence is a substantial increase in transition-state stabilization for both reactions in LeuRS by enforcing coupled behavior on the two catalytic signatures, as discussed further in the next section and the DISCUSSION.

**Figure 6.**
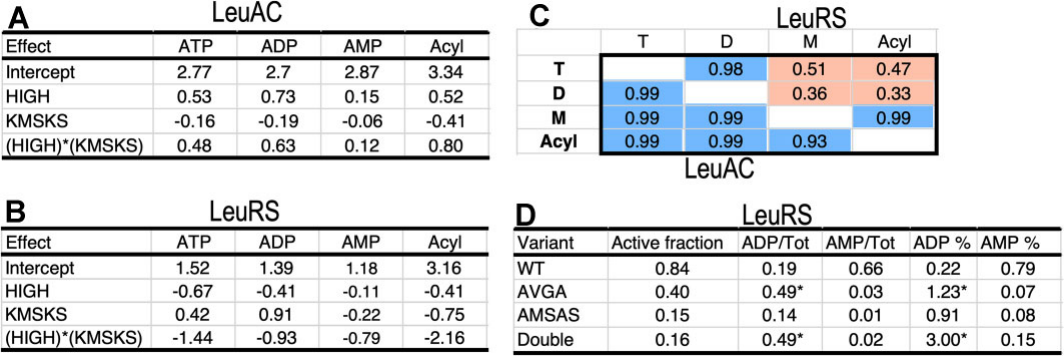
Consistent mutational impact on leucine activation and tRNA^Leu^ acylation. (**A**) Main effects and coupling energy between HVGH and KMSKS signatures for each of the reactions measured by AST and by tRNA^Leu^ aminoacylation by LeuAC. (**B**) A similar table for effects on the same reactions by LeuRS. (**C**) Correlation coefficients between the four, three element column vectors (i.e. excluding the intercepts) for the attributed free energies in A and B. LeuRS correlations are in the upper triangular matrix; LeuAC values are in the lower triangular matrix. Blue denotes near identical vectors; red denotes non-colinear vectors. Supplementary Figure S6 provides a scatterplot matrix of the full set of these values. (**D**) Burst sizes for the three reactions measured by AST for full-length LeuRS. The last two columns give the percentages of the total ATP consumption as ADP and AMP. Note that wild type LeuRS produces mostly AMP, consistent with the correlation between AMP production and acylation in C.

Thermodynamic cycle parameters built from Δ*G*^‡^*k*_chem_ values from time courses of the three nucleotides plus acylation illustrate how mutations affect all four rate constants (Figure 6). The main effects of wild-type HVGH and KMSKS signatures and their two way interaction are essentially identical in the LeuAC urzyme (Figure 6A, C). Mutational changes also impact both steps of the reaction by full-length LeuRS consistently (Figure 6B, C). Those for AMP production and tRNA^Leu^ aminoacylation by LeuRS are nearly colinear (Figure 6B). However, an important distinction for LeuRS emerges in Figure 6C. Only AMP production by full-length LeuRS has the same sensitivity to mutations as does aminoacylation. In contrast, ATP consumption and ADP production exhibit similar mutational profiles that both respond favorably to the AMSAS mutation (i.e. ΔG^‡^*k*_chem_ > 0).

Products of the internal first-order reactions in the first round of catalysis also distribute differently in full-length LeuRS and the LeuAC urzyme. The ratio, M/D, of AMP to ADP produced (Supplementary Figure S4A; *R*^2^ = 0.93; *P* ≤ 0.0001) is inversely proportional to the presence of the native KMSKS sequence. Thus, increased ATP consumption associated with LeuAC variants lacking the wild-type lysine residues is also associated with lower amounts of the expected product, AMP. In contrast, the M/D ratio in full-length LeuRS variants depends fully on both main effects (HVGH and KMSKS) and on their two-way interaction (Supplementary Figure S4B; *R*^2^ = 0.99; *P* < 0.0001), in a manner similar to the dependence of the activation free energies, Δ*G*^‡^*k*_chem_.

Figure 6C shows that contributions of the HVGH and KMSKS signatures and their two-way interactions to ATP consumption and ADP production by LeuRS are distinctly different from those contributing to AMP production and tRNA aminoacylation. In fact, their mutational profiles are closely anticorrelated to those for AMP production and acylation, as well as those for all four reactions by LeuAC (<*R*^2^> = 0.85 ± 0.1 see Supplementary Figure S5). Together with thermodynamic cycles for rates of AMP production by LeuAC and LeuRS (Supplementary Figure S3), these observations suggest an overriding distinction between catalysis of aminoacylation by full-length LeuRS and all 7 remaining variants illustrated in Figure 3B. Deletion of any contributor to the wild-type full-length LeuRS mechanism—either of the various domains missing in the urzyme or of either or both signature sequence—changes the catalytic mechanism in similar underlying manner (see again the distribution in the upper right of Figure 3B).

### Nonpolar sidechains V51 and M651 anchor both HVGH and KMSKS signatures securely into the ABD

**Figure 7.**
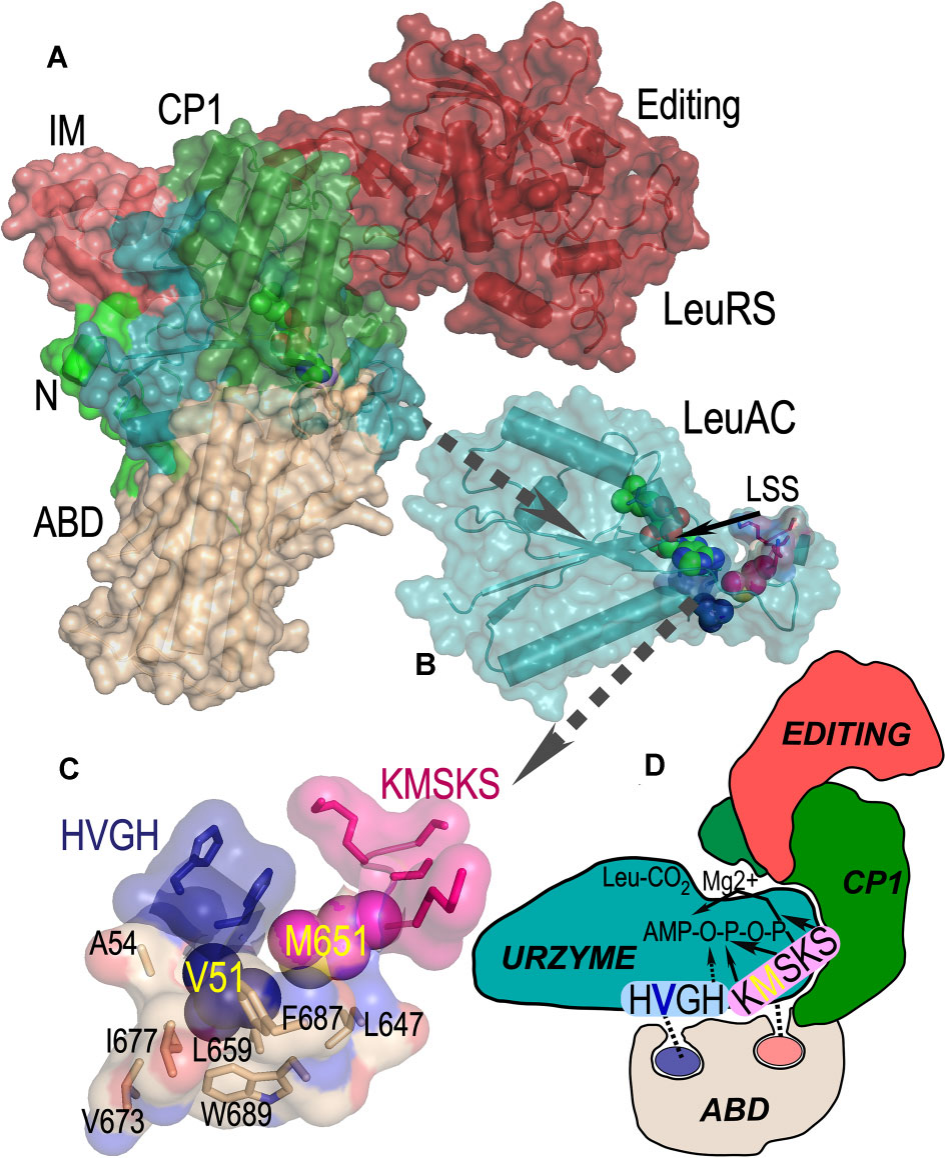
Internal mechanics of the LeuRS (B) and LeuAC (C) active sites. (**A**) Space-filling cartoon of intact LeuRS (PDB ID 1WZ2) showing the same domains with the same color scheme as in Fig. 1A. (**B**) LeuAC urzyme, LSS is a leucyl-5′sufoamyl adenosine analog of the activated amino acyl adenylate. (**C**) The ‘Enforcer’. Nonpolar side chain packing anchors V51 (blue) from the HVGH (blue) and M651 (magenta) from the KMSKS signatures firmly within the ABD. Illustrated residues participate in high statistical potential Delaunay tetrahedra surrounding the nonpolar side chains of the two signature sequences. (**D**) Cartoon highlighting the tight mechanical linkage of the two signature sequences to the ABD, and consequently to their cooperative motions relative to the urzyme.

It is worth noting that although only minimal structural data are available for any aaRS urzymes (36), neither of the hydrophobic residues in the two signature sequences appears from crystal structures of full-length Class I aaRS to be engaged in any significant nonpolar packing interactions within in LeuAC itself (Figure 7B). By contrast, a well-developed packing network of nonpolar amino acids including the two hydrophobic side chains together with residues from the ABD in full-length LeuRS anchors the valine and methionine residues within that domain (Figure 7). Delaunay tessellation and likelihood scoring (37,38) identified a homologous network in TrpRS (39), and was used here to identify residues detailed in Figure 7C. Similar networks can be identified in all full-length Class I aaRS. They appear to be both necessary and sufficient to coordinate the behavior of the two catalytic signatures, as shown schematically in Figure 7D.

## DISCUSSION

The role of cooperative interactions between active-site residues in enzyme catalysis is a recurring question that is seldom addressed directly by experimentation (40), despite well-established protocols for thermodynamic cycle analysis of combinatorial mutants (35). The thermodynamic cycle is a revealing linear transformation of the experimental transition state stabilization free energies, Δ*G*^‡^, which as noted elsewhere are additive. The alternative coordinate system highlights the magnitude and sign of individual and energetically coupled contributions to catalysis.

### Use of ^32^pα-ATP in active site titrations reveals hidden mechanistic microheterogeneity

Introducing the use of ^32^Pα-ATP in active site titration assays has expanded our appreciation of the complexity of first-order events in the first round of catalysis. The ability to track all three adenine nucleotides reveals unexpected production of small amounts of ADP even by full length LeuRS and especially in LeuRS active-site variants (Supplementary Table S2). Indeed, as discussed in RESULTS in other contexts, loss either of the additional domains or the intact catalytic apparatus in the active site is sufficient to dramatically alter the flow of chemical free energy during amino acid activation.

The equivalence between ATP consumption and the sum of ADP and AMP burst sizes, combined with the similarity of first-order rates for the three processes by LeuAC implicate similar catalytic machinery in both ADP and AMP production. The KMSKS sequence in full-length Class I aaRS is involved with transient binding of the PP_i_ leaving group of ATP (see, for example, the effects of removing PPi from the free energy surface traversed during catalysis by TrpRS (41)). A more extensive discussion of this question is in Supplementary §IV.

### Emergent coupling deepens evidence for the authenticity of urzyme catalysis

We cannot overlook the obvious conclusion that the contrast between the uncoupled behavior of the two catalytic signatures in LeuAC and their strong coupling by the ABD and CP in full length LeuRS reinforces the authenticity of both LeuAC (12) and TrpAC (25) catalysis. The evolution of intramolecular coupling shown here therefore materially strengthens the argument that aaRS urzymes represent valid experimental models for the ancestral assignment catalysts that originally enabled nature to mine the immense functional diversity represented by proteins (7).

### The LeuAC studies complement and extend earlier thermodynamic cycle analyses of TyrRS and TrpRS mechanisms

In the present case the coupling between catalytic signatures might, in principle, arise solely from the sequence differences far from the active site on the newly created molecular surface of LeuAC, rather than from the removal of the CP and anticodon-binding domains. We cannot envision any practical way to rule this possibility out, as the native sequences lead to insoluble aggregates of LeuAC. Prior studies comparing TrpAC with TrpRS substantially strengthen confidence in the interpretation outlined here, which implicate domain movement and intramolecular communication in the enhanced function of the full-length enzyme.

Class I aminoacyl-tRNA synthetase enzymes (aaRS) afford several examples of how thermodynamic cycle analysis probes structure-function relationships. First and Fersht showed that the effects of mutating residues in the KMSKS loop were localized quite specifically along the reaction profile to destabilize the ground state pre-transition state and stabilize the transition state for tyrosine activation by TyrRS (20). The KMSKS signature breaks the synergistic binding of amino acid and ATP in the pre-transition state (20–23).

Destabilization by the KMSKS signature of the transition states for both activation and aminoacylation in LeuRS appears to be inconsistent with that observation. A plausible rationale for this apparent discrepancy is that the behavior in TyrRS also depends on interaction between the two signatures. That interpretation is especially appealing because LeuAC exhibits the opposite dependence: the wild-type KMSKS sequence itself destabilizes both transition states (Figure 5). Its catalytic contribution to both reactions becomes favorable only by virtue of the two-way interaction.

A packing motif (42) called the D1 switch (39) links the two β-strands and α-helix of the first crossover connection to the N-terminal β-strand of the second crossover connection of the Rossmann dinucleotide-binding domain, creating a conformational transition state that mediates shear during relative domain motion of the ABD and CP and imposing discrete pre-transition and products conformations in TrpRS (41,43,44) and likely in other Class I aaRS. Combinatorial mutagenesis of four residues from the D1 switch, assayed with both Mg^2+^ and Mn^2+^ established a five-dimensional thermodynamic cycle. That cycle revealed that the packing motif, which is ∼20 Å from the active-site metal, is nevertheless coupled by –6.2 kcal/mol to the catalytic function of the metal (26). That contribution nearly equals the full catalytic contribution, –6.4 kcal/mol, of the metal measured by assaying metal-free TrpRS (45). A complementary, modular thermodynamic cycle consisting of TrpRS, its urzyme, TrpAC and TrpAC plus either the CP insert (i.e. the intact catalytic domain) or the ABD showed that both CP and the ABD alone actually reduced the activity of TrpAC in aminoacylation of tRNA^Trp^, but that both together restored full activity, a contribution of ∼5 kcal/mol—nearly the entire contribution of Mg^2+^ (25). Finally, both the combinatorial mutagenesis (24) and modular cycles (25) also implicate the corresponding energetic coupling in amino acid specificity.

Coupling energies between D1 switch residues and Mg^2+^ in transition-state stabilization are comparable to those between the TrpRS ABD and CP domains (24–27). The minimum action path connecting successive structures along the TrpRS structural reaction profile implies that repacking D1 switch residues in the conformational transition state is rate-limiting between pre-transition state and products domain configurations (41,43,44). These fragmentary experimental vignettes consistently suggest that a central function of the ABD and CP domains is to impose coordinated motion on components of the active site, such that they all assemble simultaneously into a catalytically active, discriminating configuration (24,25).

### Experimental exploration of the time domain

The thermodynamic cycles comparing LeuRS to its putative ancestral urzyme form in Figures 3–5 extend experimentation into the evolutionary time domain (46–53). In this manner we identify quantitatively the explicit gain of function induced in Class I leucyl-tRNA synthetase by the acquisition of the connecting peptide 1 (CP) insertion and anticodon-binding (ABD) domains. The new data reported here reinforce the picture outlined in previous paragraphs. In LeuAC, the absence of the two larger domains leaves the two catalytic signatures uncoordinated, contributing in unexpected ways to transition state stabilization. In full length LeuRS, on the other hand, although neither signature alone can enhance transition-state stabilization, their combination, in the context of the two additional domains, furnishes substantial catalytically productive synergy. That we now have identified much the same phenomena we observed for TrpRS, the smallest Class I aaRS in one of the largest as well as the smallest, Class I aaRS suggests generality in the superfamily. The superfamily-wide conservation of the ‘enforcer’ packing motif illustrated in Figure 7C, in which high SNAPP potential (37,38) hydrophobic clusters anchor the sole hydrophobic side chains of each catalytic signature, coordinating their motion reinforces its generality.

### Successively acquired CP and anticodon-binding domains may have had distinct, less obvious selective advantages

We have argued that aaRS of both Classes began as 46-residue polypeptides called ‘protozymes’ that accelerated the rate of amino acid activation ∼10^6^-fold (54). That hypothesis gained support from validation by Tamura's group (55). Thus, it is possible that the HxGH signature has roots in the protozyme. Class I aaRS urzymes, however, are more than twice as big (∼130 residues), and the KMSKS signature must have roots in that subsequent stage. The selective advantage of the KMSKS signature likely contributed to the acquisition of the second half of urzymes, which accelerated the rate of activation by 10^3^-fold over that for the protozyme.

As noted previously (25), neither CP nor the ABD enhanced either specificity or catalytic rate enhancement of the TrpRS urzyme. This prompts the question of how nature selected either domain except in the unlikely case that the urzyme acquired both simultaneously. The AST assays performed to assess the fraction of active catalysts in each sample suggest a possible resolution of that conundrum. The proportion of total ATP usefully converted to AMP production by LeuAC (0.21) is roughly 25% that (0.8; Figure 6D) observed for LeuRS. If the CP domain stabilizes closed, productive forms of the urzyme so that it used ATP more efficiently, that would certainly have given it a selective advantage, even without imposing the coupling between the two signatures. This hypothesis is experimentally testable.

### Significant rate acceleration by early polypeptide catalysts did not require sophisticated amino acid side chains

The LeuAC AVGA variant (<Δ*G*^‡^> = 1.9 kcal/mol) is quantitatively more active than the same variant of the full-length LeuRS (<Δ*G*^‡^> = 2.1 kcal/mol). That brings us to what is perhaps the most consequential implication from the data in Figures 2–4. The overall rate enhancements observed for amino acid activation by aaRS urzymes excerpted from two Class I (TrpRS (6,7,14), LeuRS (12)) and one Class II aaRS (HisRS (10,25)) represent ∼60% of the overall transition-state stabilization free energy observed for the corresponding full length aaRS. How much of that must we attribute to the precise orientation of specific amino acid side chains in the active site?

Our data suggest that the surprising answer to that question is: apparently very little, if any of it. Indeed, the superiority of the AVGA LeuAC mutant means that the two histidine residues in that highly conserved signature actually reduce the rate of aminoacylation unless they are coupled to the KMSKS sequence by the additional domains. Moreover, the AMSAS mutant is almost as active as the WT LeuAC. These observations are consistent with the previous mutational analysis of the TrpAC urzyme, where mutation of an aspartate at the N-terminus of the C-terminal α-helix of the second crossover connection in the Rossmann fold reduces activity by ∼200-fold in TyrRS but increases activity ∼25-fold in the TrpRS urzyme.

We previously noted that secondary structural; i.e. backbone interactions in Class I and II urzymes could account for tRNA (56,57) and amino acid (58) substrate selection (7). Together with our previous evidence that the TrpAC urzyme may be a catalytically active molten globule (36), these results substantiate the possibility that substantial catalytic rate enhancement does not require precise orientation of specialized amino acid side chains. It now seems that primordial protein catalysts achieved high rate enhancements and a modicum of substrate specificity largely via physical properties of the active-site pocket that depend largely on backbone interactions that do not require specific amino acid side chains.

That conclusion lends a strong experimental basis for Wong's coevolution theory for the emergence of the genetic code and its coevolution with metabolic pathways necessary to synthesize amino acids that were not produced in abundance by prebiotic geochemistry (16,17).

## Supplementary Material

gkad590_Supplemental_File

## Data Availability

JMP is a product of the SAS corporation, Research Triangle, NC, USA. All rate data are provided in Supplementary Tables, are accessible from The Protein Databank, or upon request from the author.

## Notes

Present address: Jessica J. H. Elder, National Institute of Environmental Health Sciences, Research Triangle Park, NC 27709, USA.
