# Supplementary material for: Domain acquisition by class I aminoacyl-tRNA synthetase urzymes coordinated the catalytic functions of HVGH and KMSKS motifs
Source: Nucleic Acids Res. 2023 Jul 20;51(15):8070–84. doi: 10.1093/nar/gkad590 (PMC10450160; doi:10.1093/nar/gkad590)
Supplement: gkad590_Supplemental_File [file gkad590_supplemental_file.pdf]

Supplemental Information:

Domain Acquisition by Class I Aminoacyl-tRNA Synthetase Urzymes Coordinated the Catalytic Functions of HVGH and KMSKS Motifs

Guo Qing Tang, Jessica J. H. Elder[1], Jordan Douglas[2], and Charles W. Carter, Jr, Department of Biochemistry and Biophysics, University of North Carolina, Chapel Hill, NC 27599-7260

[1] Present address: National Institute of Environmental Health Sciences, Research Triangle Park, NC 27709 ORCID ID: 0000-0002-1896-0339
[2] Department of Physics, The University of Auckland, New Zealand

I. *Experimental design matrices*

Table S1. Design matrix for single-turnover kinetic assays to determine the fraction (n) of active molecules. The first four columns are the estimated parameters from Eqn. (1). Columns 11-17 are binary entries specifying independent variables.

Variant	C	A	k_{chem}	k_{cat}	k_{chem}/k_{cat}	ΔG_{kchem}	ΔG_{kcat}	$\Delta Gk_{chem}/k_{cat}$	ATP	ADP	AMP
**LeuAC**											
**ATP**											
WT	0.6	0.36	0.004	0.000043	96	3.26	5.96	-2.7	1	0	0
AVGA	0.55	0.36	0.015	0.000043	342	2.49	5.95	-3.45	1	0	0
AMSAS	0.57	0.38	0.005	0.000044	106	3.18	5.94	-2.76	1	0	0
AVGA_AMSAS	0.57	0.4	0.008	0.000023	321	2.89	6.31	-3.42	1	0	0
**ADP**											
WT	0.3	0.28	0.003	0.000013	252	3.4	6.67	-3.27	0	1	0
AVGA	0.34	0.26	0.019	0.000036	518	2.36	6.06	-3.7	0	1	0
AMSAS	0.35	0.3	0.004	0.000013	319	3.27	6.68	-3.41	0	1	0
AVGA_AMSAS	0.36	0.33	0.008	0.000011	706	2.86	6.74	-3.88	0	1	0
**AMP**											
WT	0.11	0.08	0.006	0.000026	243	2.99	6.24	-3.25	0	0	1
AVGA	0.1	0.1	0.009	0.000007	1266	2.8	7.03	-4.23	0	0	1
AMSAS	0.09	0.08	0.006	0.000029	221	2.98	6.18	-3.2	0	0	1
AVGA_AMSAS	0.06	0.08	0.007	0.000013	577	2.9	6.67	-3.76	0	0	1
**ATP+ADP**											
WT	0.9	0.1	0.01	2.80E-05	311	2.82	6.22	-3.4	0	0	0
AVGA	0.9	0.1	0.01	7.00E-06	1305	2.77	7.01	-4.25	0	0	0
AMSAS	0.91	0.08	0.01	0.00003	256	2.89	6.17	-3.28	0	0	0
AVGA_AMSAS	0.93	0.08	0	3.00E-06	1274	3.23	7.47	-4.23	0	0	0
**LeuRS**											
**ATP**											
WT	0.46	0.47	0.22	0.000090	2406	0.91	5.51	-4.61	1	0	0
AVGA	0.76	0.13	0.02	0.000025	807	2.30	6.26	-3.96	1	0	0
AMSAS	0.87	0.06	0.13	0.000105	1234	1.21	5.42	-4.21	1	0	0
AVGA_AMSAS	0.85	0.04	0.14	0.000044	3244	1.16	5.94	-4.79	1	0	0
**ADP**											

WT	0.15	0.08	0.06	0.000094	643	1.66	5.49	-3.83	0	1	0
AVGA	0.24	0.13	0.01	0.000007	2102	2.53	7.06	-4.53	0	1	0
AMSAS	0.12	0.05	0.13	0.000102	1248	1.22	5.44	-4.22	0	1	0
AVGA_AMSAS	0.14	-0.04	0.14	0.000039	3591	1.16	6.00	-4.85	0	1	0
**AMP**											
WT	0.40	0.40	0.33	0.000018	18816	0.66	6.48	-5.83	0	0	1
AVGA	0.01	0.02	0.14	0.000006	24131	1.16	7.13	-5.97	0	0	1
AMSAS	0.01	0.01	0.12	0.000004	33578	1.25	7.42	-6.17	0	0	1
AVGA_AMSAS	0.01	0.03	0.24	0.000004	56473	0.84	7.32	-6.48	0	0	1
**ATP+ADP**											
WT	0.60	0.40	0.33	0.000018	18817	0.66	6.48	-5.83	0	0	0
AVGA	0.14	-0.04	0.14	0.000039	3591	1.16	6.00	-4.85	0	0	0
AMSAS	0.99	0.01	0.11	0.000004	26074	1.30	7.32	-6.02	0	0	0
AVGA_AMSAS	0.99	0.01	0.15	0.000003	45209	1.13	7.48	-6.35	0	0	0

Table S2. Relative activities of LeuAC and LeuRS mutants in the first round of catalysis. Values in the table are taken from n-values determined by active-site titration. ADP/TOT and AMP/TOT are the fractions of the two nucleotides observed; ADP % and AMP % are normalized to a total of 1.0 to give the proportion of each nucleotide produced in each AST experiment.

LeuAC	WT	AVGA	AMSAS	AVGA*AMSAS
Active fraction	0.64 ±0.04	0.67 ± 0.10	0.67 ± 0.06	0.69 ± 0.03
ADP/Tot	0.49	0.50	0.54	0.57
AMP/Tot	0.16	0.17	0.14	0.13
ADP %	0.77	0.75	0.80	0.83
AMP %	0.25	0.25	0.21	0.18

LeuRS	WT	AVGA	AMSAS	AVGA*AMSAS
Active fraction	0.84 ± 0.08	0.40 ± 0.13	0.15 ± 0.09	0.16 ± 0.13
ADP/Tot	0.19	0.30	0.14	0.15
AMP/Tot	0.66	0.03	0.01	0.02
ADP %	0.22	0.76	0.91	0.92
AMP %	0.79	0.07	0.08	0.15

Table S3. Design matrix for first-round rates, k_{chem} for leucine activation transition-state stabilization free energies for the four LeuAC variants, together with codes for the three independent variables of the thermodynamic cycles.

Variant	k_{chem}	ΔG_{kchem}	HVGH	KMSK	HVGH*KMSKS	FULL
**LeuAC**						
**ATP**						
WT	0.004	3.26	1	1	1	0
AVGA	0.015	2.49	0	1	0	0
AMSAS	0.005	3.18	1	0	0	0
AVGA_AMSAS	0.008	2.89	0	0	0	0
**ADP**						
WT	0.003	3.4	1	1	1	0
AVGA	0.019	2.36	0	1	0	0
AMSAS	0.004	3.27	1	0	0	0
AVGA_AMSAS	0.008	2.86	0	0	0	0
**AMP**						
WT	0.006	2.99	1	1	1	0
AVGA	0.009	2.8	0	1	0	0
AMSAS	0.006	2.98	1	0	0	0
AVGA_AMSAS	0.007	2.9	0	0	0	0
**ATP+ADP**						
WT	0.01	2.82	1	1	1	0
AVGA	0.01	2.77	0	1	0	0
AMSAS	0.01	2.89	1	0	0	0
AVGA_AMSAS	0	3.23	0	0	0	0
**LeuRS**						
**ATP**						
WT	0.22	0.91	1	1	1	1
AVGA	0.02	2.30	0	1	0	1
AMSAS	0.13	1.21	1	0	0	1
AVGA_AMSAS	0.14	1.16	0	0	0	1
**ADP**						
WT	0.06	1.66	1	1	1	1
AVGA	0.01	2.53	0	1	0	1
AMSAS	0.13	1.22	1	0	0	1
AVGA_AMSAS	0.14	1.16	0	0	0	1
**AMP**						
WT	0.33	0.66	1	1	1	1
AVGA	0.14	1.16	0	1	0	1
AMSAS	0.12	1.25	1	0	0	1
AVGA_AMSAS	0.24	0.84	0	0	0	1
**ATP+ADP**						
WT	0.33	0.66	1	1	1	1
AVGA	0.14	1.16	0	1	0	1
AMSAS	0.11	1.30	1	0	0	1
AVGA_AMSAS	0.15	1.13	0	0	0	1

Table S4. Design matrix with aminoacylation rates and transition-state stabilization free energies for the eight LeuRS variants, together with codes for the three independent variables.

Catalyst	k(/mole/sec[1]	ΔG‡(kcal/mole)	KMSKS	HVGH	HVGA*KMSKS	FULL
LeuAC_WT	0.0085	2.82	1	1	1	0
LeuAC_WT	0.0081	2.85	1	1	1	0
LeuAC_WT	0.0056	2.78	1	1	1	0
LeuAC_WT	0.0067	2.67	1	1	1	0
LeuAC_WT	0.0051	2.83	1	1	1	0
LeuAC_AVGA	0.0193	2.04	1	0	0	0
LeuAC_AVGA	0.0339	1.71	1	0	0	0
LeuAC_AVGA	0.0358	1.68	1	0	0	0
LeuAC_AVGA	0.0390	1.92	1	0	0	0
LeuAC_AVGA	0.0383	1.93	1	0	0	0
LeuAC_AMSAS	0.0063	3.00	0	1	0	0
LeuAC_AMSAS	0.0065	2.98	0	1	0	0
LeuAC_AMSAS	0.0088	2.80	0	1	0	0
LeuAC_AMSAS	0.0067	2.67	0	1	0	0
LeuAC_AMSAS	0.0057	2.77	0	1	0	0
LeuAC_AMSAS	0.0068	2.66	0	1	0	0
LeuAC_AVGA+AMSAS	0.0188	2.35	0	0	0	0
LeuAC_AVGA+AMSAS	0.0057	3.06	0	0	0	0
LeuAC_AVGA+AMSAS	0.0067	2.97	0	0	0	0
LeuAC_AVGA+AMSAS	0.0119	2.33	0	0	0	0
LeuAC_AVGA+AMSAS	0.0053	2.81	0	0	0	0
LeuAC_AVGA+AMSAS	0.0079	2.57	0	0	0	0
FL-LeuRS_WT	0.3216	0.71	1	1	1	1
FL-LeuRS_WT	0.3173	0.72	1	1	1	1
FL-LeuRS_WT	0.3011	0.75	1	1	1	1
FL-LeuRS_AVGA	0.0168	2.58	1	0	0	1
FL-LeuRS_AVGA	0.0423	2.03	1	0	0	1
FL-LeuRS_AVGA	0.0811	1.65	1	0	0	1
FL-LeuRS_AMSAS	0.0872	1.64	0	1	0	1
FL-LeuRS_AMSAS	0.1385	1.37	0	1	0	1
FL-LeuRS_AMSAS	0.1549	1.30	0	1	0	1
FL-LeuRS_AVGA+AMSAS	0.4131	1.03	0	0	0	1
FL-LeuRS_AVGA+AMSAS	0.2465	1.33	0	0	0	1
FL-LeuRS_AVGA+AMSAS	0.3175	1.18	0	0	0	1

[1] Acylation rates have been corrected by dividing by both fractional activity of the respective catalyst and the fractional activity of substrate tRNA.

II. Regression coefficients as a linear transformation of the experimental data

We previously described in some detail (1,2) the use of multiple regression methods to perform the tedious calculations involved in higher-order thermodynamic cycle analysis of enzyme kinetic data. That discussion included a detailed calculation of activation free energies associated with the various edges of the 5-dimensional hypercube formed by TrpRS directed mutants I4V, F26L, Y33F, and F37I assayed with both Mg^{2+} and Mn^{2+}. The regression model in that case reproduced the values obtained by manually subtracting the experimental free energies along each unique edge. The correlation coefficient between values obtained in that way to those estimate for the 5-dimensional linear regression model was 0.98, and the differences were localized to the approximate range $-0.5<\beta_i<0.5$ where they are poorly defined experimentally.

Full factorial design leads to exactly equal numbers of variant catalysts and parameters to estimate as potential coefficients, β_i, β_{ij}, etc., in the corresponding regression model. It is useful to recognize that this means the thermodynamic cycle formalism provides an alternate reference frame for the experimental data points. The coordinate axes in the two frames—the designated mutations and the complete set of main effects and higher-order interactions—are just different ways to consider the same experimental vectors. The regression β coefficients furnish a more efficient presentation of the classic thermodynamic cycles and hence an important basis for interpreting experimental data. They represent estimates for the intrinsic effects of each site in a combinatorial mutagenesis and all significant higher-order energetic coupling between mutated sites.

The designated mutations in the present case are given in the various design matrices in Tables S1, S2, and S3, where they are associated with free energies obtained from experimental rate measurements. Regression coefficients are plotted as histograms in Fig. 6 of the main text. To clarify how one converts back and forth from one representation to the other Figs. S1 and S2 compare the representations for activation free energies for the two-way cycle for $\Delta G^{\ddagger}k_{chem}$ from AST and the three-way thermodynamic cycle of $tRNA^{Leu}$ acylation activities (AVGH, AMSAS mutations in full-length LeuRS and LeuAC urzyme). Least squares multiple regression converts from activation free energies derived from rate data to intrinsic and higher-order effects by estimating the β-coefficients (Fig. S1B, S2B).

Those coefficients can then be used to re-calculate values for the activation free energies using Eqn [2] (Fig. S1C, S2C).

A

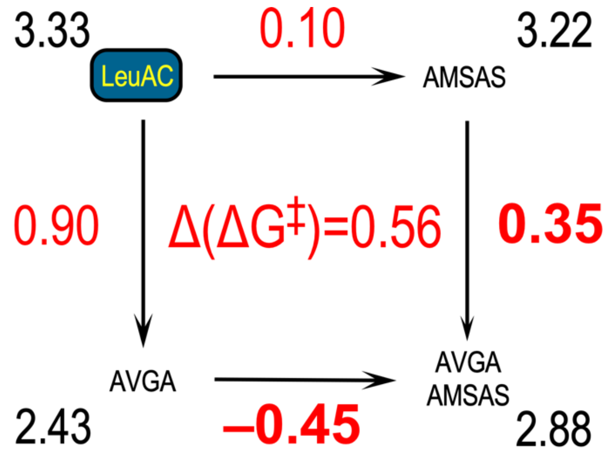

B

Term	β	σ	β/σ	Prob>ltl
Intercept	2.88	0.05	53.9	<.0001*
HIGH	0.35	0.08	4.6	0.0097*
KMSKS	-0.45	0.08	-6.0	0.0040*
HIGH*KMSKS	0.56	0.11	5.2	0.0065*

C

Variant	Intercept	KMSKS	HVGH	KMSKS*HVGH	SUM	<ΔG‡kchem>
WT	2.875	-0.45	0.35	0.56	3.33	3.33
AVGA	2.875	-0.45	0.00	0.00	2.43	2.43
AMSAS	2.875	0.00	0.35	0.00	3.23	3.22
AVGA_AMSAS	2.875	0.00	0.00	0.00	2.88	2.88

Figure S1. Thermodynamic cycle for LeuAC $\Delta G^{\ddagger}k_{chem}$. **A**. Classical presentation. Mean values of $\Delta G^{\ddagger}k_{chem}$ are at the corners, associated with each variant. Values of $\Delta(\Delta G^{\ddagger}k_{chem})$ along each edge are calculated in the direction of the arrows. All values are in kcal/mole. Values for the left and bottom edge are in bold because the regression model in **B** have been estimated without recentering the cross term, so that it is easier to follow the computation of the free energies for each mutant in **C**. **B.** Regression model estimated without recentering the cross term. Values for the intrinsic effects of the two signatures illustrated in Fig. 2A of the main text were computed with a recentered cross term (KMSKS – 0.5)*(HVGH–0.5) and are less biased estimates. For that reason, only the values in bold face in **A** match values of the β coefficients for the intrinsic effects. The R^2 value for the eight experimental measurements is 0.98. **C.** Computation of average values observed experimentally for $\Delta G^{\ddagger}k_{chem}$. Components of Eqn. [2] are presented in successive columns, and the SUM is equal to the calculated $\Delta G^{\ddagger}k_{chem}$. R^2 between the final two columns is 0.999.

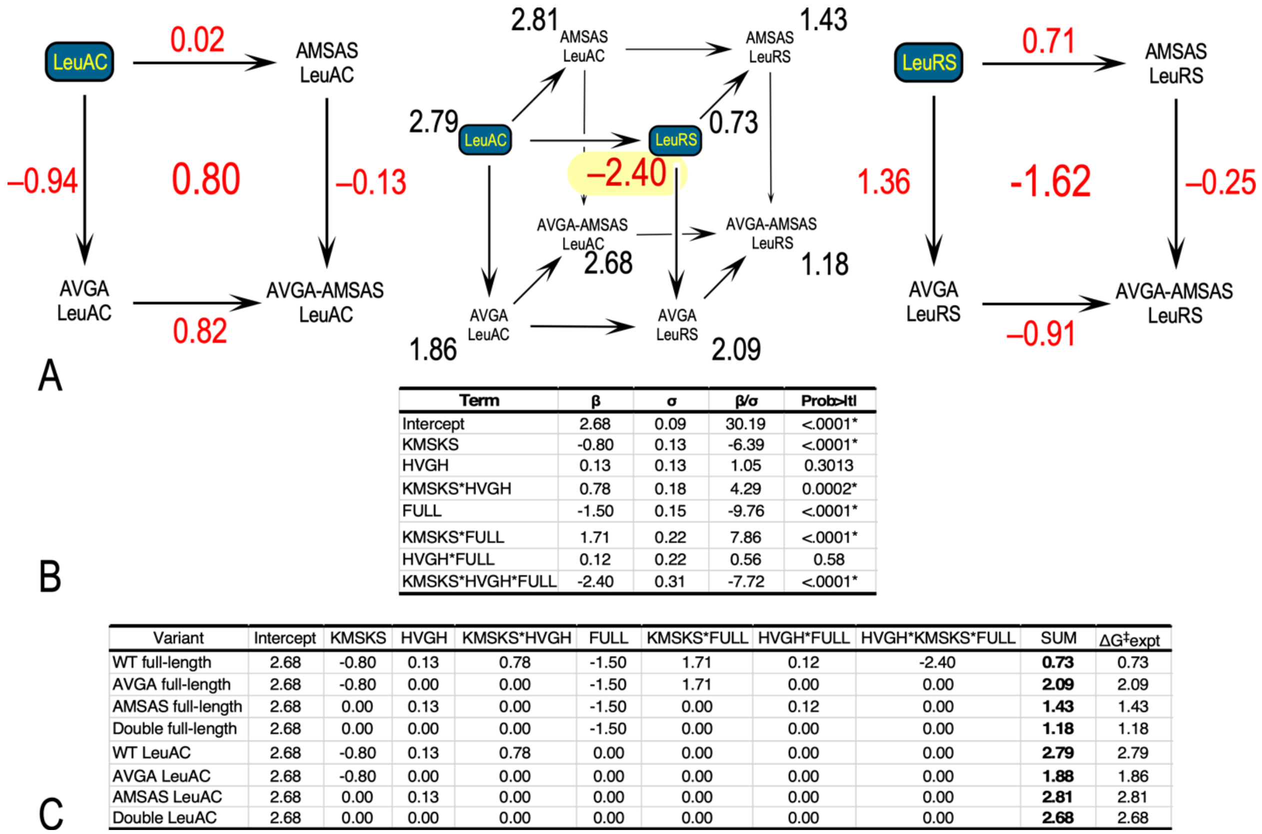

A

B

Term	β	σ	β/σ	Prob>ltl
Intercept	2.68	0.09	30.19	<.0001*
KMSKS	-0.80	0.13	-6.39	<.0001*
HVGH	0.13	0.13	1.05	0.3013
KMSKS*HVGH	0.78	0.18	4.29	0.0002*
FULL	-1.50	0.15	-9.76	<.0001*
KMSKS*FULL	1.71	0.22	7.86	<.0001*
HVGH*FULL	0.12	0.22	0.56	0.58
KMSKS*HVGH*FULL	-2.40	0.31	-7.72	<.0001*

C

Variant	Intercept	KMSKS	HVGH	KMSKS*HVGH	FULL	KMSKS*FULL	HVGH*FULL	HVGH*KMSKS*FULL	SUM	$\Delta G^{‡}expt$
WT full-length	2.68	-0.80	0.13	0.78	-1.50	1.71	0.12	-2.40	**0.73**	0.73
AVGA full-length	2.68	-0.80	0.00	0.00	-1.50	1.71	0.00	0.00	**2.09**	2.09
AMSAS full-length	2.68	0.00	0.13	0.00	-1.50	0.00	0.12	0.00	**1.43**	1.43
Double full-length	2.68	0.00	0.00	0.00	-1.50	0.00	0.00	0.00	**1.18**	1.18
WT LeuAC	2.68	-0.80	0.13	0.78	0.00	0.00	0.00	0.00	**2.79**	2.79
AVGA LeuAC	2.68	-0.80	0.00	0.00	0.00	0.00	0.00	0.00	**1.88**	1.86
AMSAS LeuAC	2.68	0.00	0.13	0.00	0.00	0.00	0.00	0.00	**2.81**	2.81
Double LeuAC	2.68	0.00	0.00	0.00	0.00	0.00	0.00	0.00	**2.68**	2.68

Figure S2. Interconvertibility of variant activation free energies and the thermodynamic cycle representation of intrinsic and higher-order interaction free energies. **A**. Classical representation of the three-dimensional thermodynamic cycle described in the text. The marginal LeuAC and LeuRS two-dimensional cycles (left and right-side faces of the central cube are displayed to either side. Corners of the cube have the mean experimental activation free energies for each variant from the combinatorial mutagenesis, together with the overall three-way interaction, $\Delta(\Delta G^{‡}_{KMSKS*HVGH*FULL})$ highlighted in yellow. The marginal cycles show individual $\Delta G^{‡}$ values for each edge in red, together with the corresponding $\Delta(\Delta G^{‡}_{KMSKS*HVGH})$ values in slightly larger typeface in the center. **B**. Regression model coefficients for the full thermodynamic cycle in the center of A. Student t-test P-values in the last column show that the intrinsic effect of HVGH and the HVGH*FULL interaction $\Delta(\Delta G^{‡})$ value are not significant, hence that the model is not overfitted. Estimates for intrinsic and low-order interactions differ somewhat from values in A for reasons discussed in the text. **C**. Table of values showing how Eqn. [2] in the main text is used to compute the calculated values matched with the observed data by least squares. Entries in successive columns represent the contribution made by each term of the regression model in B to the overall total activation free energies at the corners of the cube in A. The model (B) reproduces all by 7 % of the variation in the individual experimental data points (Table S3) and predicts the average values essentially exactly (R^2 = 0.999).

III. Centering polynomials: estimating regression coefficients in the presence of strong interactions.

The β coefficients of the regression model differ to some extent from those computed from the classical thermodynamic cycle representation (Fig. S1A, S2A). Although the highest-order interaction free energy is exactly the same, effects attributed to the intrinsic and lower-order interactions differ. The reasons for this are not obvious and have to do with the fact that when two factors interact significantly, it matters where along the interaction the main effects are estimated. For this reason, statistics programs like JMP do this estimation at the mean value of each of the independent variables that participate in the interaction (3). See, for example https://community.jmp.com/t5/Discussions/estimates-in-multipule-regression/td-p/10963. That process is called 'centering the polynomials' because each of the marginal quantities—intrinsic

effects and low-order interactions are evaluated at the midpoint of the corresponding interaction. Thus, because an interaction is mathematically a product of lower order terms, it is a polynomial.

Interaction coefficients represent the degree to which the dependent variable at one value of an independent variable changes when measured at different levels of a second independent variable. Hence, it gives the magnitude of energetic coupling between different factors varied in the experimental designs used here. It makes no difference whether the independent variables are continuous or discrete, as they are here. Similar statements apply to higher-order interactions, in which coupling between two variables changes when measured at different levels of a third independent variable, and so on. Without additional investigation of the nature of the coupling, those coupling energies are hard to interpret. JMP (2) provides several visual tools to aid interpretation (Fig. S3).

The important distinction between LeuAC and LeuRS is how the KMSKS*HVGH interaction changes during the evolutionary maturation of the contemporary enzyme. The JMP Profiler illustrates this coupling interactively. Fig. 2A represents how the KMSKS*HVGH interaction behaves for LeuAC and LeuRS, which are the two levels of the FULL factor. In LeuAC, the intrinsic effect of wild-type KMSKS leads to higher activity (i.e., negative slope); that of wild-type HVGH leads to lower activity (i.e., positive slope). In the full-length LeuRS, both intrinsic effects lead to higher catalytic activity and the two slopes are inverted (Fig. 2A).

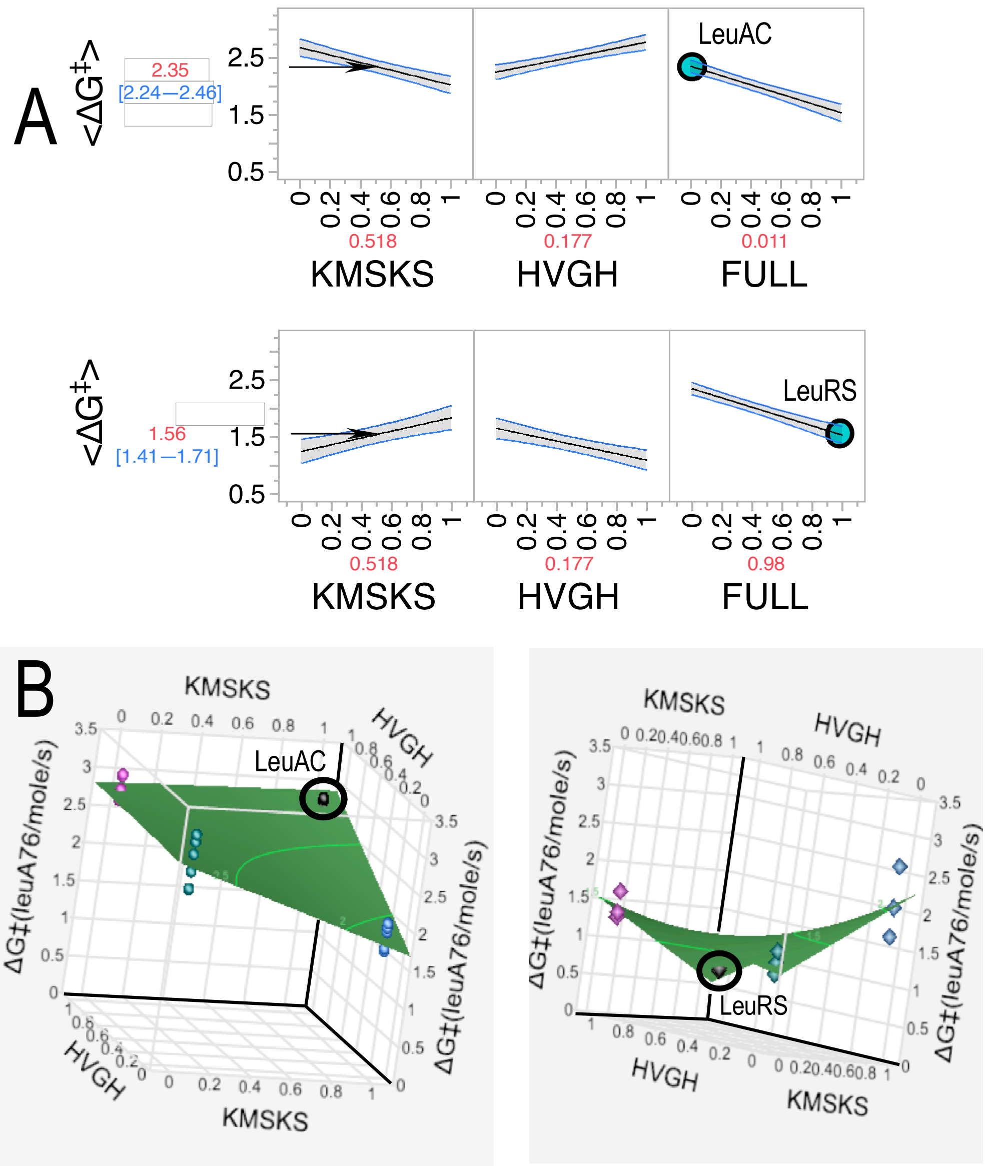

Figure S3. The HVGH*KMSKS interaction. **A**. Interactive Profiler plots of the contributions of the KMSKS and HVGH to the activation free energy in LeuAC and LeuRS. The vertical axis in all plots is the activation free energy for aminoacylation of $tRNA^{Leu}$ in kcal/mole. The horizontal axes represent the independent variables corresponding to the respective labels. The third panel (FULL) represents the difference between urzyme (LeuAC) and full-length (LeuRS) enzyme. Comparison of the top and bottom rows of panels shows how the main effects of the two catalytic sigatures change between the two catalysts. **B.** Surface plots of the KMSKS*HVGH interactions in the two catalysts. All axes have the same scale in both cases. The catalyst with wild type catalytic signatures is located in the rear corner of both plots to highlight the overall improvement in the catalyzed rate for LeuRS. The LeuAC surface is slightly concave, the LeuRS is convex with respect to the diagonal from wild type to double mutant (rear to front). Data points plotted are the triplicate experimental measurements.

IV. Supplemental mechanistic insights derived from AST experiments.

Use of ^{32}Pα-ATP to follow all three nucleotides in active site titration assays brings unexpected insight into previously obscure processes, highlighted by the unexpected production of ADP, which does not result from the canonical displacement of pyrophosphate in amino acid activation. The amplitudes of AMP produced by the variants studied in the main manuscript depend on the total amount of ATP consumed and the proportion of that consumed ATP that produced ADP. For the LeuAC urzyme, the amount of AMP produced is on the order of 20% for each variant whereas

the amount of ADP produced is on the order of 80 %. For full-length LeuRS, the amount of AMP produced is small (~ 20%) for WT LeuRS and quite large (~80-90 %) for all three of its mutants (Supplementary Table S2).

Both catalysts show strong relations between the proportion of AMP produced and the mutated sites (Fig. S2). However, as is also true for both transition-state free energies estimated from kinetic rates, the productive proportion of ATP consumed depends only on the presence of the KMSKS signature in LeuAC, whereas LeuRS shows the strong cooperativity of the two signatures, which is roughly twice the contribution of either signature's main effect. The distribution of different reaction products thus also reflects the factorial design in much the same way as the reaction kinetics.

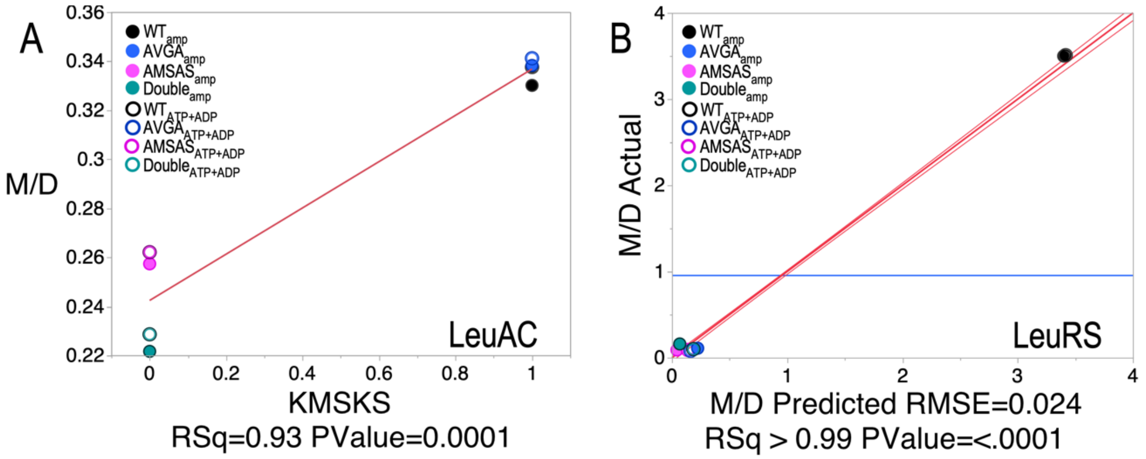

Term	β	σ	β/σ	Prob>ltl
Intercept	0.24	0.007	33	<.0001
KMSKS	0.09	0.010	9	0.0001

Term	β	σ	β/σ	Prob>ltl
Intercept	-0.77	0.011	-70	<.0001*
HVGH	1.71	0.013	135	<.0001
KMSKS	1.71	0.013	135	<.0001
HVGH*KMSKS	3.41	0.025	135	<.0001

Figure S4. The proportion of ATP consumed that is represented by AMP formation by LeuAC and LeuRS depends in quite different ways on the mutated sites. **A**. In LeuAC, the dependence is solely on the KMSKS signature and is entirely unrelated to the HVGH signature. **B**. In LeuRS the dependence is on both the main effects of HVGH and KMSKS signatures and their two-way interaction, which exceeds both main effects by a factor of ~2. The statistical significance of this comparison is strengthened by the fact that two estimates are available for the AMP produced. Solid circles represent AMP production measured by the appearance of that nucleotide on TLC plates; Open circles represent the difference between ATP consumed and ADP produced and is estimated by adding the ADP produced back to the ATP consumption curve. Student t-test P-values therefore probably only indicate experimental errors.

V. Catalytic contributions of HVGH and KMSKS sequences to AMP production during amino acid activation.

AST assays performed with ^{32}PαATP also provide $\Delta G^{\ddagger}$ values for AMP production, from which thermodynamic cycles provide energetic contributions for the two signatures and their catalytic synergy. Data summarized in Fig. 4C of the main text show that the catalytic energy profiles for ATP consumption and ADP production differ significantly from those for AMP production. Those thermodynamic cycle β coefficients are shown in Fig. S5.

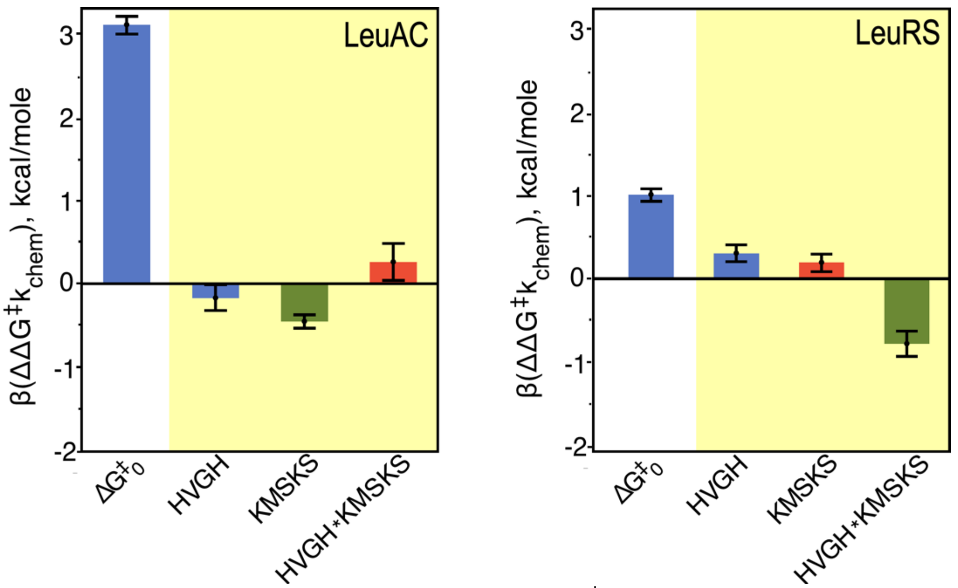

Figure S5. Thermodynamic cycles for the activation free energies, $\Delta G^{\ddagger}k_{chem}$ for AMP formation by LeuAC and LeuRS. Yellow panels highlight significant differences between the behavior of all variants and the wild type LeuRS. The LeuAC pattern is essentially the same as those of all variants excepting the WT LeuAC in both amino acid activation and $tRNA^{Leu}$ aminoacylation. The full-length LeuRS behaves in a unique manner.

VI. Detailed correlations between thermodynamic cycle parameters for all reactions observed for LeuRS and LeuAC.

Thermodynamic cycle analyses for each of the four reactions analyzed in the main text reveal considerable consistency across both reactions, The similarities and differences are summarized in the scatterplot matrix in Fig. S6.

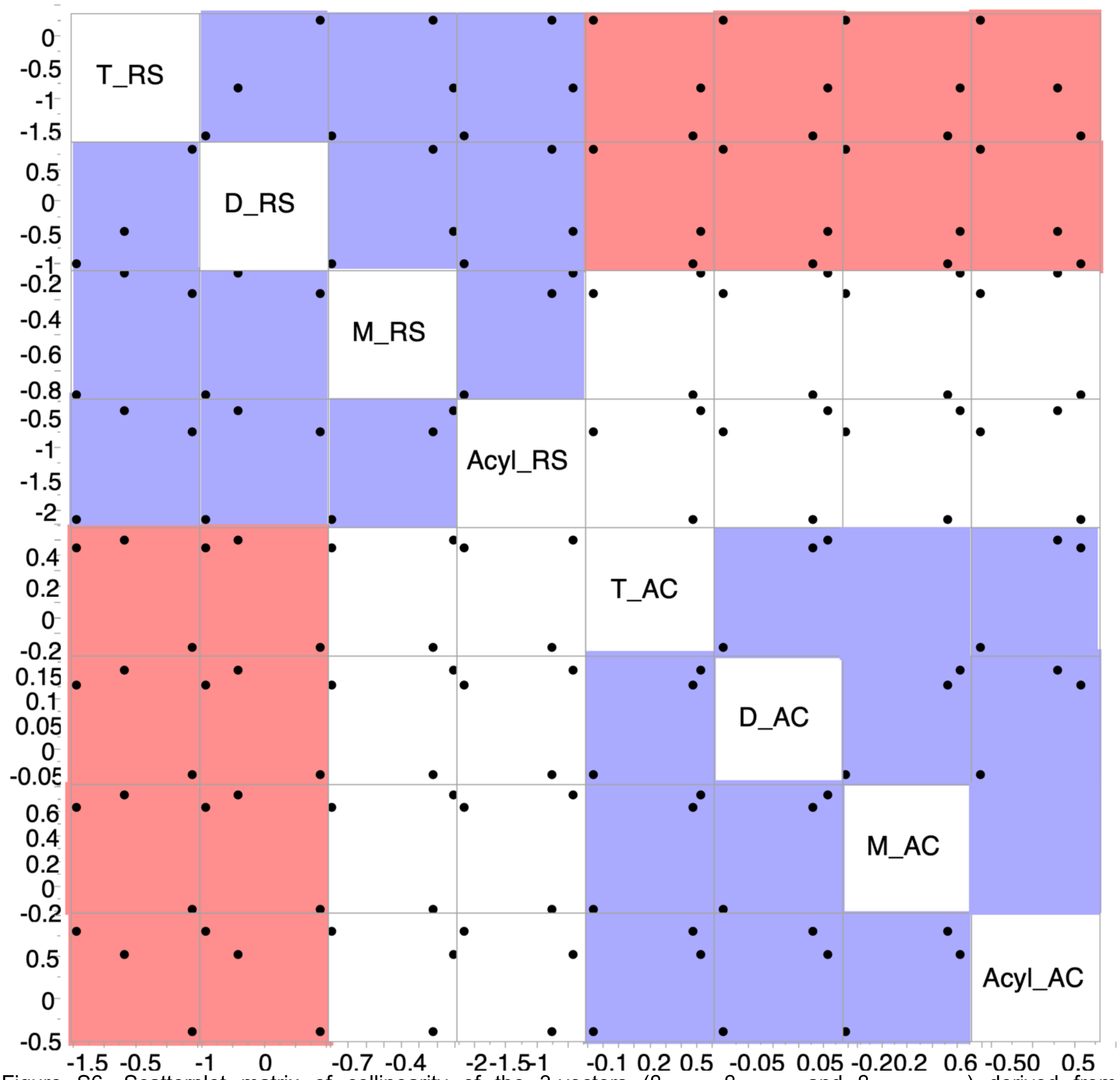

Figure S6. Scatterplot matrix of collinearity of the 3-vectors (β_{HVGH}, β_{KMSKS}, and $\beta_{HVGH*KMSKS}$) derived from thermodynamic cycle analysis of $\Delta G^{\ddagger}k_{chem}$ for ATP consumption (T_), ADP production (D_), AMP production (M_), and $\Delta G^{\ddagger}k_{acyl}$ for $tRNA^{Leu}$ aminoacylation (Acyl_) by LeuRS and LeuAC. Individual reactions are listed in the diagonal squares. Blue squares denote positive correlations with R^2 values ranging from 0.88–0.99). Red squares denote anticorrelations with R^2 values ranging from 0.72-0.99. A subset of these values appears in Fig. 6C of the main text.

References

1. Weinreb, V., Li, L. and Carter, C.W., Jr. (2012), *Structure*, Vol. 20, pp. 128-138SI.
2. SAS. (2021). V.16.0.0 ed. SAS Institute, Cary NC, Cary, NC.
3. Julian. (2015), JMP User Community, Vol. 2023, pp. Video describing why centering polynomials is useful in multiple regression.
